# Raman-based PAT for VLP precipitation: systematic data diversification and preprocessing pipeline identification

**DOI:** 10.3389/fbioe.2024.1399938

**Published:** 2024-05-31

**Authors:** Annabelle Dietrich, Robin Schiemer, Jasper Kurmann, Shiqi Zhang, Jürgen Hubbuch

**Affiliations:** Institute of Process Engineering in Life Sciences, Section IV: Biomolecular Separation Engineering, Karlsruhe Institute of Technology (KIT), Karlsruhe, Germany

**Keywords:** Raman spectroscopy, virus-like particles, protein precipitation, chemometrics, preprocessing, pipeline optimization, partial least squares regression, process monitoring

## Abstract

Virus-like particles (VLPs) are a promising class of biopharmaceuticals for vaccines and targeted delivery. Starting from clarified lysate, VLPs are typically captured by selective precipitation. While VLP precipitation is induced by step-wise or continuous precipitant addition, current monitoring approaches do not support the direct product quantification, and analytical methods usually require various, time-consuming processing and sample preparation steps. Here, the application of Raman spectroscopy combined with chemometric methods may allow the simultaneous quantification of the precipitated VLPs and precipitant owing to its demonstrated advantages in analyzing crude, complex mixtures. In this study, we present a Raman spectroscopy-based Process Analytical Technology (PAT) tool developed on batch and fed-batch precipitation experiments of Hepatitis B core Antigen VLPs. We conducted small-scale precipitation experiments providing a diversified data set with varying precipitation dynamics and backgrounds induced by initial dilution or spiking of clarified *Escherichia coli*-derived lysates. For the Raman spectroscopy data, various preprocessing operations were systematically combined allowing the identification of a preprocessing pipeline, which proved to effectively eliminate initial lysate composition variations as well as most interferences attributed to precipitates and the precipitant present in solution. The calibrated partial least squares models seamlessly predicted the precipitant concentration with *R* ^2^ of 0.98 and 0.97 in batch and fed-batch experiments, respectively, and captured the observed precipitation trends with *R* ^2^ of 0.74 and 0.64. Although the resolution of fine differences between experiments was limited due to the observed non-linear relationship between spectral data and the VLP concentration, this study provides a foundation for employing Raman spectroscopy as a PAT sensor for monitoring VLP precipitation processes with the potential to extend its applicability to other phase-behavior dependent processes or molecules.

## 1 Introduction

Virus-like particles (VLPs) as non-viral vectors have emerged as class of protein nanoparticles for vaccines, surface antigen presentation and targeted delivery (Qian et al., 2020). VLPs are composed of viral protein subunits mimicking the structure of the virus they descend from (Chackerian, 2007; Zeltins, 2013). Since VLPs lack the active viral genome, they are considered safer than other viral nanoparticles such as adeno-associated virus or lentivirus (Chackerian, 2007; Nooraei et al., 2021). The recombinant production of VLPs in eukaryotic or prokaryotic cells (Kushnir et al., 2012) lead to process material of high variability and complexity, which is even more pronounced for intracellular production as a lysis step is required (Effio and Hubbuch, 2015). Hence, in early downstream processing (DSP), the removal of the majority of process-related host cell impurities is particularly advised (Effio and Hubbuch, 2015). Native precipitation was found highly selective for VLPs and is induced by addition of a precipitation agent, using either polyethylene glycol (Tsoka et al., 2000; Koho et al., 2012) or ammonium sulfate (AMS) (Kim et al., 2010; Zahin et al., 2016; Kazaks et al., 2017). Whereas steric exclusion is typically associated with polyethylene glycol (Iverius and Laurent, 1967; Poison, 1977), the effect of protein surface charge predominates for precipitation induced by AMS as sulphate is strongly kosmotropic (Curtis et al., 1998). The protein surface charge is in turn affected by its structural properties, which may impact the precipitation behaviour (Hämmerling et al., 2017) and hence, the precipitation agent concentration required for precipitation.

The Hepatitis B core Antigen (HBcAg) VLP, a recombinantly produced internal protein capsid of the Hepatitis B virus, is subject to intensive research for diverse medical purposes (Cooper and Shaul, 2005; Porterfield et al., 2010; Klamp et al., 2011; Mobini et al., 2020; Moradi Vahdat et al., 2021; Petrovskis et al., 2021; Hassebroek et al., 2023). In previous capture studies, the AMS-induced precipitation of HBcAg VLPs was found highly selective (Valentic et al., 2022), while co-precipitation of impurities was only observed for higher AMS concentrations (Wegner and Hubbuch, 2022). Moreover, the required AMS concentration for precipitation was found to be dependent on the surface properties comparing HBcAg VLPs with and without foreign epitopes rather than on internal structural differences comparing various lengths of nucleic acid binding sites (Hillebrandt et al., 2020; Valentic et al., 2022). In the context of process development, the conventional centrifugation-based HBcAg VLP capture has been replaced by an innovative setup involving fed-batch precipitation and diafiltration-based wash and redissolution steps (Hillebrandt et al., 2020). This development aimed to establish a size-based platform process applicable to various protein nanoparticles. However, the development of a standardized platform process for protein nanoparticle DSP remains uncommon, primarily due to their extensive variety, which presents significant challenges in processing (Moleirinho et al., 2020).

To be able to operate such a capture platform process for different protein nanoparticles, the implementation of Process Analytical Technology (PAT) for process monitoring is crucial. Since 2004, the FDA has underscored the importance of real-time process monitoring for its role in enhancing process understanding, ensuring process robustness, and guaranteeing product safety within the biopharmaceutical industry (FDA, 2004; Rathore et al., 2010; Glassey et al., 2011). Besides the optical spectroscopic techniques ultraviolet-visible (UV/Vis) and infrared (IR) spectroscopy, Raman spectroscopy coupled with chemometrics has found extensive applications in monitoring various processes for biopharmaceutical products, including raw material testing (Li et al., 2010), cell culture (Abu-Absi et al., 2011; Berry et al., 2015; Golabgir and Herwig, 2016), chromatography (Feidl et al., 2019; Rolinger et al., 2021; Wang et al., 2023), filtration (Rolinger et al., 2023), freezing (Roessl et al., 2014; Weber and Hubbuch, 2021), or formulation (Wei et al., 2022). For VLPs in particular, recent studies demonstrated the real-time monitoring of a baculovirus cultivation for the production of rabies VLPs (Guardalini et al., 2023a; b; c) as well as the cross-flow filtration-based polishing operations such as dis- and reassembly of the HBcAg-VLPs (Rüdt et al., 2019; Hillebrandt et al., 2022). Despite the broad applicability of spectroscopic methods in biopharmaceutical processing, their application to precipitation processes is rather unexplored. While multiple studies have used near-infrared (NIR) spectroscopy for monitoring of acid (Sun et al., 2020) or alcohol precipitation (Li et al., 2014; Huang and Qu, 2018; Sun et al., 2018), Raman spectroscopy has only been used to track a precipitation polymerization reaction (Meyer-Kirschner et al., 2016) and monoclonal antibody precipitation (Lohmann and Strube, 2021). A major challenge when working with precipitate-containing solutions is the turbidity of the media, which was reported to affect the overall signal intensity of Raman spectra (Van Brink et al., 2002; Sinfield and Monwuba, 2014; Meyer-Kirschner et al., 2016). The loss in signal intensity may either be corrected by isolated Raman bands (Sinfield and Monwuba, 2014; Meyer-Kirschner et al., 2016) or be directly used for correlation to a target quality attribute (Meyer-Kirschner et al., 2016). Similarly, Zelger et al. (2016) have used a turbidity sensor for monitoring protein precipitation, foregoing the molecular information that could have been attained through the utilization of spectroscopic techniques.

Among the available spectroscopic techniques, Raman spectroscopy stands out for its selectivity to monitor multiple quality attributes (Santos et al., 2018; Wei et al., 2022; Wang et al., 2023) as well as its capability to analyze complex solutions such as fermentation broths (Abu-Absi et al., 2011; Berry et al., 2015; Goldrick et al., 2020) or clarified cell lysates (Genova et al., 2018; Hassoun et al., 2018). However, Raman spectroscopy comes with a set of challenges, such as low signal intensity and strong background interferences requiring extensive data preprocessing and model optimization (Rinnan et al., 2009; Bocklitz et al., 2011; Gautam et al., 2015). Due to this sensitivity, undesired data variability can arise by changes in process material or sample processing, compromising comparability and accuracy in the recorded data. Popular approaches for the removal of undesirable systematic variation include signal corrections, filtering, or variable selection (Rinnan et al., 2009; Bocklitz et al., 2011; Mehmood et al., 2012). Most commonly, baseline (He et al., 2014; Zhang et al., 2020), background (Wold et al., 1998; Trygg and Wold, 2002; Boelens et al., 2004), and scatter (Martens et al., 2003; Kohler et al., 2008; Liland et al., 2016) correction methods are employed to eliminate interferences caused by contaminating species or scattering effects such as fluorescence or Mie scattering. Since the direct subtraction of reference spectra as a basic approach for background correction might result in insufficient- or over-corrections (Boelens et al., 2004), multiple approaches have been proposed to identify and remove the undesirable background variation (Lorber, 1986; Wold et al., 1998; Trygg and Wold, 2002; Ben-David and Ren, 2005). Among those the most commonly applied algorithm is the orthogonal projection to latent structures (OPLS) as proposed by Trygg and Wold (2002) effectively removing systematic background variation not correlated with the target quality attribute (Passos et al., 2010; Abdallah et al., 2019). To further optimize model performance, additional steps like spectral cropping, derivative filtering, or filter-based variable selection may be used (Gautam et al., 2015; Santos et al., 2018). In principle, the sequence of preprocessing operations, or preprocessing pipeline, should be designed to remove irrelevant variations from the recorded data and provide the multivariate model with consistent information to enable robust and accurate real-time monitoring. Finally, a careful evaluation of the extracted information by the preprocessing pipeline and the selected multivariate model is demanded by the regulatory agencies (FDA, 2015). For linear models, such as partial least squares (PLS), multiple approaches such as variable importance in projection (VIP)-based feature importance (Mehmood et al., 2012) have been proposed to perform this quality check.

In this study, we present a PAT approach based on Raman spectroscopy for a precipitation-based capture process of VLPs. Based on multiple small-scale precipitation experiments with material from various lysate batches and spiking materials, we evaluate the suitability of Raman spectroscopy for analyzing precipitate-containing lysates. By introducing a pretreatment strategy for the UV/Vis reference measurements and identifying a suitable preprocessing pipeline for Raman spectroscopy data, we demonstrate the reduction of variance in both the Raman and the reference data and enable the prediction of the precipitated VLPs from precipitate-containing lysates. We systematically combine numerous preprocessing operations and isolate the effects of the included operations on model performance. Eventually, we transfer this approach to a fed-batch precipitation process, ensuring its adaptability and effectiveness across different operational paradigms.

## 2 Materials and methods

### 2.1 Experiments

#### 2.1.1 Virus-like particles

In this study, Cp149, a C-terminally truncated wild-type HBcAg protein (Zlotnick et al., 1996) was used, for which the plasmid was kindly provided by Prof. Adam Zlotnick (Indiana University, US). The intracellular expression of Cp149 in *Escherichia coli* *(E. coli)*, cell harvest, cell lysis, and lysate clarification were conducted as previously described by Hillebrandt et al. (2020). Clarified lysate solutions (hereinafter termed lysate) were thawed, 0.2 μm-filtered, directly used raw or conditioned by dilution or spiking depending on the precipitation experiment, and adjusted to 0.25% (v/v) polysorbate 20.

To systematically diversify the lysate for the precipitation experiments, different lysate batches were used and initial lysate conditioning was performed by dilution and spiking for eight batch experiments B1-B8 and three fed-batch experiments F1-F3. Raw lysates of two lysate batches were used for batch experiments B1 and B4, while a third lysate batch served as lysate for all fed-batch experiments. Dilution of the lysates introduced further changes in initial lysate concentration (B2-B3, F1, F3), which is expressed as volume percent lysate content and was simply measured as 280 nm absorbance (A280_L_) due to the lysates complexity.

To establish a foundation for systematic spiking, the initial composition of raw lysate was first estimated in pre-experiments (data not shown) to estimate a VLP content despite the lysates complexity. For this pre-experiment, selective VLP precipitation was conducted by adjusting raw lysate to 1.1 M AMS and the supernatant was analyzed by reference analytics. The difference between the ultraviolet (UV) absorbance at 280 nm of the initial lysate (A280_L_) and the volume-corrected supernatant after precipitation (A280_S_) account for the absorbance of selectively precipitated VLP (A280_VLP_). The calculated ratio A280_VLP_/A280_L_ expressing the estimated VLP content resulted in approximately 10% VLP present in the raw lysate. This ratio served as foundation for experimental spiking design under the assumption of purified VLP- or host-cell protein (HCP)-enriched spiking material. While the estimated VLP content with 10% remained constant using raw lysate (B1, B4) or during lysate conditioning by dilution (B2-B3, F1, F3) or spiking with salt (B8), the estimated VLP content was systematically varied by VLP- or HCP-spiking (B5-B7, F2). Note that spiking the lysates also resulted in a form of dilution; however, the primary focus was here on systematically adjusting the lysate composition. The final lysate conditioning settings of lysate batch, lysate content, and estimated VLP content are summarized for all batch experiments B1-B8 and fed-batch experiments F1-F3 in Tables 1, 2, respectively.

**TABLE 1:** Overview of the eight batch precipitation experiments B1-B8 comprising parameters for initial lysate conditioning and the final, curve fit-derived, apparent concentration of precipitated VLPs.

Batch	Condition	Lysate conditioning			Precipitated VLP
		Batch	Lysate content	Estimated VLP content	g L^-1^
		-	% (v/v)	% (A280_VLP_/A280_L_)	
B1	raw	lysate 1	100	10	4.35
B2	dilution		52.5	10	2.96
B3	dilution		35	10	2.06
B4	raw	lysate 2	100	10	3.92
B5	VLP spike		50	15	4.15
B6	VLP spike		50	13	4.16
B7	HCP spike		31	6	1.49
B8	NaCl spike		80	10	4.69

**TABLE 2:** Overview of the three fed-batch precipitation experiments F1-F3 comprising parameters for initial lysate conditioning and fed-batch processing. Lysate batch 3 was used for all experiments.

Fed-batch	Condition	Lysate conditioning		Processing	
		Lysate content	Estimated VLP content	Time	Feed rate
		% (v/v)	% (A280_VLP_/A280_L_)	min	mL min^-1^
F1	dilution	43	10	30	0.16
F2	VLP spike	32	23	30	0.16
F3	dilution	45	10	20	0.30

More details on preparation and composition of buffers, solutions, and VLP- and HCP-enriched spiking material are stated in Supplementary Section  1.1.

#### 2.1.2 Batch precipitation experiments

Conditioned lysate varied in lysate batch, concentration or composition across the eight batch VLP precipitation experiments as summarized in Table 1. For each experiment, eleven solutions were prepared in 1,040 μL-scale with precipitant concentrations in the range of 0–1.2 M AMS. In general, the solution compositions were designed to maximize the lysate content under the limitation of 4 M AMS stock solution to set the highest target AMS concentration of 1.2 M, resulting in 728 μL lysate and 312 μL 4 M AMS stock solution. Maintaining 728 μL lysate to prevent a dilution effect within the experiments in batch mode, the remaining 312 μL was composed of proportionally 4 M AMS stock solution and ultrapure water to cover the other precipitant concentrations below 1.2 M AMS. Solutions were incubated at 22°C on a thermo-shaker ThermoMixer Comfort (Eppendorf, Hamburg, DE) at 500 rpm for 30 min. For Raman measurements, 200 μL samples of the turbid precipitate solutions were taken and immediately analyzed. To enable measuring the particulate-free supernatants, the remaining turbid precipitate solutions were centrifuged at 12,000 rcf for 8 min in a Pico 17 tabletop centrifuge (Thermo Fisher Scientific Inc., Waltham, US). The supernatants were analyzed via Raman spectroscopy and UV reference analytics.

#### 2.1.3 Fed-batch precipitation experiments

The setup for fed-batch VLP precipitation consisted of a stirred reservoir equipped with a Minipuls 3 peristaltic pump (Gilson, Villiers le Bel, FR) for a 4 M AMS feed. For online monitoring, a second peristaltic pump (Gilson), a SLS-1500 flow meter (Sensirion), and a flow cell for Raman measurements were connected with PEEK capillaries with an inner diameter of 0.25 mm in an on-line loop. The on-line loop flow rate was adjusted to 1.4 mL/min. Fed-batch precipitation experiments were performed with 12 mL conditioned lysate. The lysate and processing conditions are listed in Table 2. During fed-batch precipitation, 200 μL samples were taken at each time step of in total 30 time steps, from which 22 selected samples were centrifuged at 12,000 rcf for 3 min facilitating UV reference analytics of the supernatants. For each time step, lysate and AMS content were calculated considering drawn sample volume and a mean feed volume, which was based on a mean feed flow rate considering total AMS amount and feed density.

#### 2.1.4 Raman spectroscopy

The Raman BioReactor BallProbe inserted in the FlowCell Adapter (both MarqMetrix, Seattle, US) was connected to a HyperFlux^TM^ PRO Plus 785 with the software SpectralSoft 3.2.6 (Tornado Spectral Systems, Toronto, CA). All measurements were performed in the flow cell with a laser power of 495 mW and with 50 acquisitions per spectrum. For precipitate and supernatant samples, the exposure time was set to 275 ms and 185 ms, respectively, as with precipitate being present, a higher exposure was necessary to achieve a similar intensity level due to the dampening effect of the precipitates in solution. The entire spectral range from 200 to 3300 cm^-1^ was recorded with a spectral resolution of 1 cm^-1^. For each of the eleven AMS conditions per experiment, the 50 recorded Raman spectra were averaged. For the fed-batch experiments, the 50 recorded spectra closest to the sampling time point were averaged.

#### 2.1.5 Reference analytics

All supernatant samples were analyzed via UV spectroscopy with a high performance liquid chromatography (HPLC) system equipped with a RS diode array detector and controlled by Chromeleon 6.8 (Dionex Ultimate 3000 RS, Sunnyvale, US). A 0.5 μm pre-column filter catridge (OPTI-SOLV EXP, Supelco, Bellefonte, US) but no column was installed. A buffer containing 50 mM Tris, 100 mM NaCl at pH 8.0 was used as mobile phase with a flow rate of 50 μL min^-1^ and an injection volume of 20 μL. Samples were diluted 100-fold in triplicates in lysis buffer and UV spectra in the wavelength range from 220 to 400 nm were recorded.

### 2.2 Data analysis and computation

Data analysis and computation was performed in Python 3.8.

#### 2.2.1 Reference data pretreatment

Several pretreatments steps for the UV reference data were conducted in order to transform the scattered data into reliable reference data for the chemometric modeling. The pretreatment comprised scatter correction, scaling, precipitation curve estimation, outlier removal, and a final conversion to the apparent precipitated VLP concentration. Scatter correction for the 280 nm peak areas (A280) was performed to remove nucleic acid contributions according to Porterfield and Zlotnick (2010), whereas AMS content-dependent scatter correction was not required due to the 100-fold sample dilution.

Each batch experiment consisted of A280-triplicates for 11 AMS conditions, resulting in 33 data points. To reduce the effect of outliers in the data within one experiment, a robust least squares fit of the precipitation curve using the Boltzmann function was performed. This model-based approximation assumes that the precipitation can be divided into three distinct phases, namely an initial plateau until the onset of the precipitation, a sigmoidal decrease, and a final plateau once all target molecules have been precipitated. In particular, the optimization problem was set up as Eq. 1

$$\min_{x}=0.5\overset{N}{\underset{i=1}{\sum }}\delta {\left(f\left(c_{\text{AMS},i},x\right)-y_{i}\right)}^{2}$$ (1)

where *y*_*i*_ denote the discrete UV measurements at a certain concentration of AMS *i* and *f* (*c*_AMS,*i*_, **x**) being the Boltzmann function given as Eq. 2

$$f\left(c_{\text{AMS},i},x\right)=\frac{x_{2}-x_{4}}{1+\exp\left(-\left(x_{1}c_{\text{AMS},i}+x_{2}\right)\right)}+x_{3}.$$ (2)

The parameter vector **x** is defined as ${\left(x_{1},x_{2},x_{3},x_{4}\right)}^{T}$ . By employing the non-linear transformation of the squared residuals *z*, a smooth approximation of the absolute value loss by $\delta \left(z\right)=2\left(\sqrt{\left(1+z\right)}-1\right)$ was generated to effectively reduce the impact of outliers. To generalize the problem where the threshold between inliers and outliers is different from 1, the formula $\hat{\delta }\left(r^{2}\right)=C^{2}\delta \left({\left(r/C\right)}^{2}\right)$ was used with *C* being a scaling factor that determines the threshold between outliers and inliers in the data set. In our case, *C* was set to 5 for robust parameter estimation across all batch and fed-batch experiments.

The generated Boltzmann fits were used to remove outliers in each experiment. The distribution of the residuals between the Boltzmann curves and the original measurements were analyzed. Data points with residuals outside of the interval [*p*_0.25_ – 1.5*IQR*; *p*_0.75_ + 1.5*IQR*] were excluded from the data set with the interquartile range *IQR* being defined as *p*_0.75_ − *p*_0.25_ (Wilcox, 2005) and *p*_0.25_ and *p*_0.75_ the 25% and 75% percentiles.

Within one experiment, the conversion to apparent precipitated VLP concentration was done by subtracting the minimum observed A280 mean from all data points, taking the absolute values, and scaling by HPLC device parameters and the theoretical extinction coefficient of the VLP at 280 nm of 1.764 L g^−1^ cm^−1^ as provided by the ProtParam tool (Gasteiger et al., 2005). This procedure assumes that solely VLP is precipitating under given precipitation conditions as discussed in Wegner and Hubbuch (2022) and the absorption is only caused by VLP in solution. Under consideration of other HCPs and nucleic acids in the solution, this procedure is biased and hence the derived VLP content is termed apparent precipitated VLP concentration.

#### 2.2.2 Spectral data processing

Spectral data processing of averaged spectra was done in order to reduce unwanted differences in the collected Raman data and covered spectral outlier removal, turbidity correction, baseline correction, background correction, difference spectra, derivative and smoothing, and cropping. Multiple combinations of the listed operations were screened to find the optimal configuration. Hence, the individual operations will be explained in detail.

##### 2.2.2.1 Spectral outlier removal

Spectral outlier were removed manually based on visual inspection regarding defective spectra. Defective spectra were found for experiment B2 corresponding to conditions 1.0, 1.1 and 1.2 M AMS and occurred mainly due to sample handling and hence the inclusion of air bubbles in the flow cell.

##### 2.2.2.2 Turbidity correction

To remove interfering Raman effects caused by turbidity, the spectra were turbidity-corrected by normalization at 3299 cm^-1^.

##### 2.2.2.3 Baseline correction

All spectra were baseline-corrected using a Whittaker filter employing the adaptive smoothness penalized least squares (asPLS) according to Zhang et al. (2020) and implemented in *pybaselines* (v. 1.0.0). The *λ* parameter was determined manually by visual inspection of the estimated baseline. The optimal *λ* value of 6 × 10^−7^ was chosen based on the most consistent baseline estimation over the entire spectral range across all samples. Furthermore, a second-order difference matrix, a maximum number of iterations of 100, and a tolerance of 10^–3^ were used.

##### 2.2.2.4 Background correction

To remove the interfering Raman effects of the buffer system and the precipitating agents, two different background correction methods were evaluated. The first one which we will refer to as scaled substraction (SS) uses reference measurements of the buffer system at the respective concentration levels of AMS. The reference spectra were scaled to the Raman spectra collected in the precipitation experiments by normalization at 750 cm^-1^. As the SS method can only be applied when reference measurements are available, a second background correction method was evaluated, namely the OPLS as described in Trygg and Wold (2002). The OPLS method extracts the orthogonal systematic variation in the spectral data which is not correlated to the target quality attribute and was used as implemented in *pyopls* (v 20.02) using 3 latent variables.

##### 2.2.2.5 Difference spectra

Since only the precipitated material contributes to the changes in Raman intensity, difference spectra were computed per experiment by subtracting the first spectrum of each experiment. This is analogous to using the autozero function of other spectroscopic detectors at the beginning of the experiments. In essence, this is supposed to remove the varying background effects of the lysate matrix of which the exact composition is unknown.

##### 2.2.2.6 Derivative and smoothing

Derivative and smoothing was applied using the Savitzky-Golay filter (SGF) (Savitzky and Golay, 1964). Unless stated otherwise, the SGF was used with a second-degree polynomial and a window size of 11 as implemented in *scipy* (v. 1.11.4).

##### 2.2.2.7 Cropping

Spectra were cropped to reduce the influence of fluorescent background, scattering, buffers, and precipitant. Multiple intervals were evaluated to study the effect of individual contributions. The selected intervals for cropping of the Raman spectra were 800–1800, 1020–1800 and 1200–1500 cm^-1^, which were motivated by the exclusion of residual baseline variance, the largest sulfate contribution and the majority of buffer interference.

##### 2.2.2.8 Preprocessing pipeline

The individual preprocessing operations are used in sequence as presented above. In some cases, a differing order may be viable, however the authors decided to keep the order fixed for this study in order to maintain comparability of the different approaches. The preprocessing pipeline configuration identified as optimal for the batch data was utilized for the fed-batch experiments.

##### 2.2.2.9 Quantitative evaluation

To compare preprocessing operations quantitatively without the need to calibrate a multivariate regression model, the metric signal-to-noise ratio (SNR) was used to evaluate the correlation between individual variables with the target quantity. Here, we define the SNR as

$$S\;N\;R=\frac{Var\left(x_{\lambda }\hat{\beta }\right)}{{\hat{\sigma }}^{2}}$$ (3)

with $\hat{\beta }$ being the regression coefficients of a univariate linear model of type y = *x*_λ_ *β* + *ɛ* with normally distributed errors *ɛ* and ${\hat{\sigma }}^{2}$ being the residual variance of linear model for wavenumber *λ* according to Soch and Allefeld (2018). To calculate SNR for the Raman spectroscopy data using Eq. 3, a linear model was built for each wavenumber individually. The SNR was used to compare multiple combinations of preprocessing operations. All combinations were applied to the precipitate and supernatant data sets. A preprocessing operation is considered beneficial if the metric at a certain variable increases compared to the un-preprocessed state. Further, the variable is considered predictive if it shows high SNR in precipitate and supernatant samples among all experiments.

#### 2.2.3 Regression modelling

##### 2.2.3.1 Data splitting

To make maximum use of the recorded data, the batch data (B1-B8) were split using a nested cross-validation scheme. The eight experiments are divided into six training and two test experiments according to a *Leave-Two-Experiments-Out* cross-validation scheme which serves as the outer cross-validation. In total, this results in 8!/(2!∗6!) = 28 independent test sets on which the calibrated models are evaluated. For the inner cross-validation, the six training experiments are further rotated using a *Leave-One-Experiment-Out* cross-validation scheme to perform hyperparameter optimization in each of the iterations of the outer cross-validation. The fed-batch data were split in two training experiments (F1, F3) and one independent test experiment (F2). Here, the hyperparameters were optimized using a randomized split of the training set with eleven validation data points.

##### 2.2.3.2 Regression models

For the comparison of different combinations of preprocessing operations, two model types were evaluated as multivariate regressors for the quantification of the apparent precipitated VLP concentration, namely multiple linear regression (MLR) and PLS models as implemented in *scikit-learn* (v. 1.3.2). The different model types were selected to resolve potential differences between the working principles of these models with regard to preprocessing pipelines. For the MLR models, six wavenumbers of all observed wavenumbers were selected which were known to be protein-related but were not affected by AMS, namely 830, 850, 1241, 1314, 1341 and 1617 cm^-1^ (Spinner, 2003; Maiti et al., 2004; Rygula et al., 2013). PLS models were trained using the NIPALS algorithm (Wold et al., 2001) and the number of latent variables was optimized using a grid-search in the range of 2–10 based on the inner cross-validation. Before being passed to the regression models, all spectral data were mean-centered and column-wise scaled to unit variance. PLS models for quantification of AMS were built based on a pipeline including turbidity correction, baseline correction, difference spectra and no further optimization was performed.

##### 2.2.3.3 Error metrics

Accuracy of the calibrated models was assessed using the root mean squared error (RMSE) and the coefficient of determination *R* ^2^. For the inner cross-validation, the RMSE was calculated using the left-out experiments. When PLS models were used, the RMSE was scaled by the number of latent variables according to Wold et al. (2001). The importance of individual wavenumbers in PLS models was assessed quantitatively by VIP scores. The VIP score *v*_*j*_ (Mehmood et al., 2012) is defined as Eq. 4

$$v_{j}=\sqrt{N\overset{A}{\underset{a=1}{\sum }}\left[\left(q_{a}^{2}t_{a}^{T}t_{a}\right){\left(w_{aj}/\left\|w_{a}\right\|\right)}^{2}\right]/\overset{A}{\underset{a=1}{\sum }}\left(q_{a}^{2}t_{a}^{T}t_{a}\right)},$$ (4)

for the individual wavenumber *j* ∈ [1, *N*], where *N* denotes the total number of wavenumbers. The loading weights, the y-loadings, and the score vector corresponding to the PLS component *a* ∈ [1, *A*] are given by *w*_*a*_, *q*_*a*_, and *t*_*a*_, respectively.

## 3 Results

### 3.1 Spiking diversifies experimental data

**FIGURE 1:**
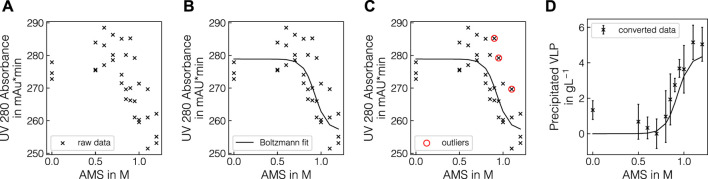
Illustration of pretreatment of the UV absorbance measurements. The UV absorbance at 280 nm is exemplarily shown over precipitant concentration for selected pretreatment steps for experiment B1. Raw triplicate UV measurements are shown as black crosses **(A)** and the fitted Boltzmann function is shown as a solid black line **(B)**. The detected outliers are represented by red circles **(C)**. The converted precipitated VLP concentrations are shown as black crosses with one standard deviation from averaging the three replicates per condition **(D)**.

**FIGURE 2:**
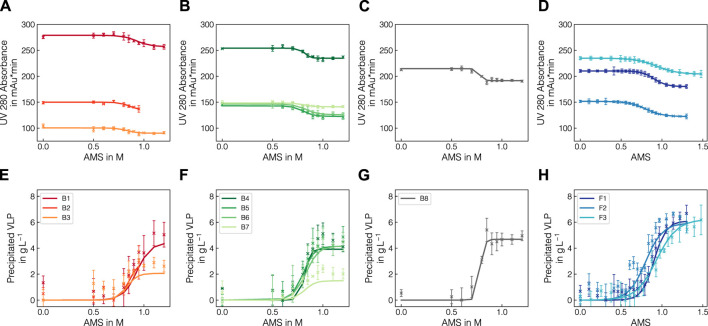
Comparison of pretreated UV absorbance data and VLP precipitation curves. Total UV absorbance at 280 nm after scatter- and outlier correction **(A–D)** and converted precipitation curves **(E–H)** are shown for batch experiments B1-B8 and fed-batch experiments F1-F3. Solid lines represent the fitted Boltzmann functions for outlier detection. The experiments are grouped according to the mode of spiking, where B1-B3, B4-B7 and B8 show the influence of lysate dilution, protein-composition regarding or salt content, respectively. For B2, the data points above 0.95 M were excluded due to defective Raman spectra in the precipitate samples.

Experiments in batch and fed-batch mode were conducted in 1 mL and 12 mL scale, respectively. To generate variance in precipitation data, multiple lysate batches and defined spiking solutions were used. In total, eight batch experiments and 3 fed-batch experiments were conducted (see Tables 1, 2) and samples, taken at different concentrations of AMS, were analyzed by Raman spectroscopy and reference analytics. To extract the apparent precipitated VLP concentration from the reference UV absorbance measurements, the UV data were treated as described in 2.2.1. The UV pretreatment procedure is schematically depicted in Figures  1A–D. Figure 1A shows the scatter-corrected UV data for experiment B1 over the precipitation range from 0 to 1.2 M AMS. To approximate the precipitation trend, a Boltzmann function which is shown in Figure 1B was fitted to the displayed data points. Based on the residuals between the Boltzmann fit and the observed data, outliers were excluded which reduced the spread of observed data as visible in Figure 1C. Finally, the UV data were converted to the apparent precipitated VLP concentration as presented in Figure 1D. The resulting outlier-corrected UV absorbance progression and the corresponding apparent VLP concentration are shown for all experiments in Figure 2. For the batch experiments, three distinct groups are illustrated, as the experimental conditions were induced by different methodologies. For each experiment, the outlier-corrected absorbance measurements are displayed with one standard deviation and the fitted Boltzmann functions. While the Boltzmann functions aligned well with the UV absorbance for all experiments, the height of the final plateau for the precipitated VLP concentration was underestimated in the case of B3 and B7. Experiments B1 to B3 are shown in Figures 2A, E and were generated by diluting the lysate material using lysis buffer. For these curves over the course of the AMS concentration, the reductions of the total UV absorbance as well as the amount of precipitated VLP indicate the reduced concentration of all components by dilution. Experiments B1 and B4 used lysate material from different batches and showed comparable total UV absorbance and precipitated amounts of VLP. Compared to B4, experiments B5-B7 (cf. Figures 2B, F) were generated by specifically enriching the VLP or HCP content in the lysate materials by adding spiking material. This resulted in varying total UV absorbance and precipitated amounts of VLP for these individual curves. While a maximum of 2 gL^−1^ of precipitated VLP was reached in B7, the remaining experiments showed precipitated amounts of VLP up to 5 gL^−1^. In contrast to B4, experiments B5-B7 varied in the range of 125 and 150  mAU total UV absorbance. In experiment B8 (cf. Figures 2C, G), the NaCl concentration was increased to 270 mM, which caused a lower UV absorbance compared to B4 which was based on the same lysate material and reached a maximum amount of precipitated VLP of over 4 gL^−1^. The final, apparent concentrations of precipitated VLPs derived from the fitted Boltzmann functions are listed in Table 1.

In the fed-batch experiments (cf. Figures 2D, H), the differences in the precipitation trends were induced by dilution, adding VLP-enriched spiking material and modulating the feeding rate of the precipitant solution (cf. Table 2). Here, similar trends to the batch experiments were observed in terms of varying UV absorbance ranges. Note that the observed data include the dilution caused by the addition of the precipitant and hence a more gradual increase in the precipitated VLP concentration is expected compared to the batch experiments. Moreover, the AMS content calculation for F3 revealed a higher mean AMS flow rate than expected, exceeding the initially chosen final concentration of 1.3 M AMS.

In summary, the generated precipitation data provide insights into the precipitation dynamics in the presence of varying backgrounds induced by the addition of spiking material or buffer solution and serve as a diverse data set for chemometric model development.

### 3.2 Raman spectra are affected by precipitant and precipitates

**FIGURE 3:**
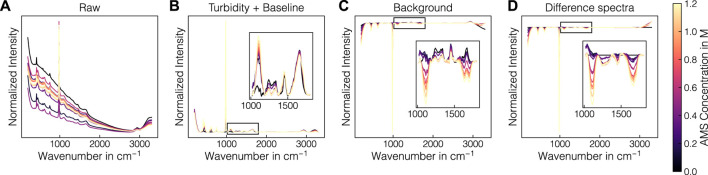
Illustration of the preprocessing of the Raman spectra. The preprocessing procedure is exemplarily depicted for experiment B1. The normalized raw Raman spectra **(A)** and selected preprocessing steps **(B–D)** are shown. The spectra are colored according to the AMS concentration with brighter colors denoting higher concentrations.

Raman spectra are commonly affected by the matrix, buffers and other components present in the sample. Before using the collected Raman data for chemometric modeling, the data require preprocessing to properly evaluate potential interferences from buffers or excipients. A schematic illustration of the effect of the employed preprocessing operations is presented in Figure 3. From left to right, the schematic pipeline shows the averaged raw Raman spectra for each sample in experiments B1, the turbidity- and baseline-corrected spectra using the 3299 cm^-1^-normalization and asPLS Whittaker filter, the SS-background-corrected spectra and the difference spectra. The combination of turbidity correction with the Whittaker filter removed turbidity effects and baseline drifts reliably over the whole spectral range (cf. Figures 3A, B). The SS-background correction subtracts the contribution of the AMS, which caused the AMS-related band at 980 cm^-1^ to become negative and the protein-related bands to show larger variations (cf. Figure 3C). Finally, the difference spectra operation sets the Raman intensity in the beginning of the experiments to 0 and caused negative bands in the wavenumber region 1200–1700 cm^-1^ (cf. Figure 3D). For a visual interpretation of the difference spectra, the reader is advised to regard positive and negative bands as substances being added and being removed from the solution, respectively. For evaluating the interferences of the precipitant, buffers or excipients, the Raman spectra were firstly treated by turbidity- and baseline correction only.

**FIGURE 4:**
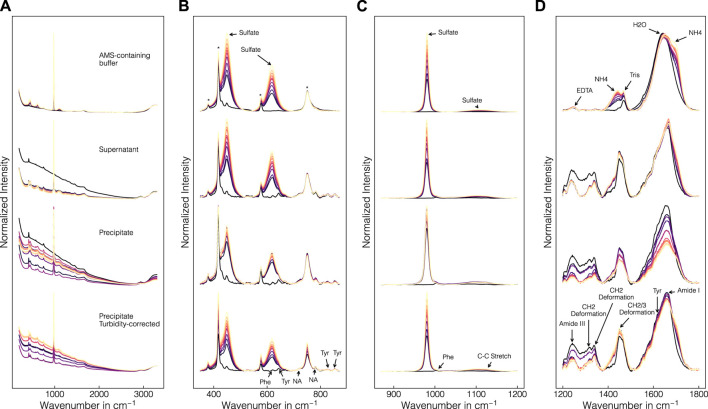
Comparison of collected Raman spectra in precipitant-containing buffer, supernatant and precipitate samples. Precipitate and supernatant data are exemplarily shown for batch experiment B1. Full raw spectra **(A)**, and baseline-corrected spectra of selected wavenumber regions **(B–D)** are illustrated. Spectra with incorporated turbidity correction are shown in the bottom row for the precipitate samples. The spectra are normalized in each row and subfigure for visual purposes and colored according to the AMS concentration with brighter colors representing higher concentrations. Wavenumber regions indicated by arrows correspond to buffer-, protein- and nucleic acid (NA)-related contributions. Sapphire bands are marked with an asterisk.

Figure 4 presents a comparison of the collected Raman spectra in precipitant-containing buffer samples, supernatant samples, precipitate samples from batch experiment B1 without and with turbidity correction (cf. Section 2.2.2) from top to bottom. Figure 4A shows the raw Raman spectra and Figures 4B–D show the baseline-corrected data within selected wavenumber regions. In the raw spectra (cf. Figure 4A), different baseline effects are visible between all sets of spectra with the precipitate data showing the strongest baseline variations. Baseline variations in the precipitate data were less pronounced for the turbidity-corrected spectra. At low concentrations of AMS, the baseline increased while at high AMS concentrations, the baseline decreased again. This effect was accompanied by the increasing contributions of AMS in the Raman spectra with the most prominent Raman band being located at 980 cm^-1^. Additionally, the increasing AMS contents are visible in the Raman bands at 450, 618 and 1106 cm^-1^ (cf. Figures 4B, C) as well as near 1435 and 1693 cm^-1^ (cf. Figure 4D), which can be attributed to sulphate and ammonium ions, respectively (Spinner, 2003; Fontana et al., 2013). Additional components of the lysis buffer which were contained in all samples can be traced to the bands at 1249 and 1470 cm^-1^ indicative for Tris and EDTA (Socrates, 2004). These bands remained roughly constant over the course of a precipitation experiment as only the AMS concentration was gradually increasing. As expected, the sapphire bands at 379, 418, 430, 450, 577 and 750 cm^-1^ (Watson et al., 1981), the band of molecular oxygen at 1556 cm^-1^ (Weber et al., 1967) and the broad water band at 1650 cm^-1^ (Spinner, 2003) remained roughly constant as well. Protein-related contributions are located between 600–880 cm^-1^ and 1200–1800 cm^-1^ with amide bands, CH_2_-deformation bands and bands of aromatic amino residues (Maiti et al., 2004; Rygula et al., 2013). Amide III and I bands are visible near 1241 and 1660 cm^-1^, respectively. Tyrosine (Tyr) bands appear at 830, 850, 1205 and 1617 cm^-1^ and the bands originating from tryptophan (Trp) and phenylalanine (Phe) appear at 1340 and 1605 cm^-1^, respectively. CH_2_-deformations are visible at 1314, 1340 and 1448 cm^-1^. While these protein-related contributions were largely unaffected by the AMS or lysis buffer induced Raman activity, the precipitant interfered with the protein-related contributions in several locations (cf. 620, 643, 1004, and 1127 cm^-1^). Tyr, Phe, and C-C stretching can be associated with these bands at 643 cm^-1^, 620 and 1004 cm^-1^, and 1127 cm^-1^, respectively (Peticolas, 1995; Maiti et al., 2004; Rygula et al., 2013). In addition to the protein-related contributions, the bands at 724 and 781 cm^-1^ are indicative for the presence of nucleic acids (Peticolas, 1995).

To study the effect of the precipitates in solution formed during the precipitation experiment, the baseline-corrected Raman spectra from precipitate and supernatant samples were compared. In general, similar bands are visible in both the precipitate and the supernatant spectra. The protein bands showed a decreasing trend with increasing AMS concentrations. This effect was more pronounced in the precipitate spectra compared to the supernatant spectra indicating an overall protein band-specific intensity decrease caused by the precipitates in solution. Similarly, the nucleic acid bands at 724 and 781 cm^-1^ were visibly decreasing with increasing concentrations of AMS. By introducing the turbidity correction, the overall intensity decrease was reduced in all mentioned wavenumber regions, effectively making the precipitate spectra more comparable to the supernatant spectra, especially in the bands of the nucleic acids. However, individual Raman bands were adversely effected such as the sapphire band at 750 cm^-1^, showing minor overcorrection.

### 3.3 Raman data reveal structural differences between species

**FIGURE 5:**
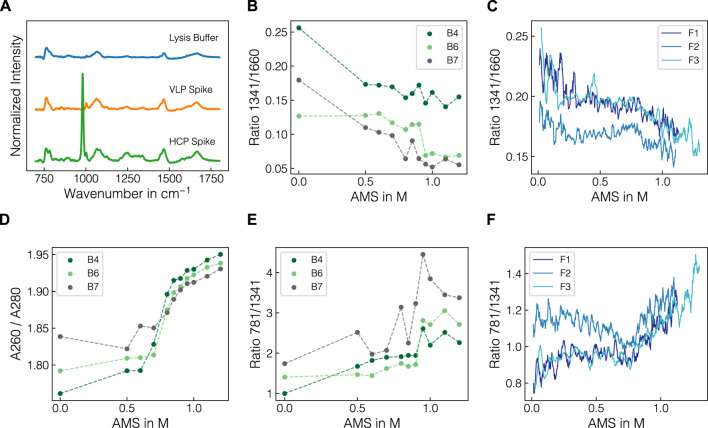
Raman spectra of the spiking solutions and comparison of selected wavenumber ratios. Normalized Raman spectra of lysis buffer and VLP- and HCP-enriched spiking materials after baseline-correction and subtraction of water blank are illustrated in **(A)**. The protein-band ratio 1341/1660 is depicted over the course of AMS for batch experiments B4, B6, B7 differing in the protein composition of the lysate batch **(B)** and all fed-batch experiments **(C)**. The UV absorbance-derived A260/A280 ratio **(D)** is shown alongside the band ratio 781/1341 comparing the nucleic acid-associated Raman band with the protein-associated Raman band for batch experiments B4, B6, B7 **(E)** and fed-batch experiments **(F)**. All ratios were calculated with turbidity- and baseline-corrected Raman spectral data. The ratios for fed-batch experiments were calculated using a moving average of 10 spectra to smooth the signal. Dashed lines are only shown for visual purposes.

To investigate whether structural differences between the target molecules and the other species in the lysates are observable via Raman spectroscopy, the Raman spectra from the VLP- and HCP-enriched spiking materials were compared. Normalized Raman spectra of the lysis buffer and the purified VLP- and the HCP-enriched spiking solutions are presented in Figure 5A after baseline-correction and water blank subtraction. Both solutions exhibited distinct Raman bands in the protein fingerprint region between 800 and 1800 cm^-1^ with slight differences in the shape of the most prominent peaks between 1000–1100 cm^-1^, 1200–1300 cm^-1^ and 1600–1700 cm^-1^. Additionally, the nucleic acid-related Raman band at 781 cm^-1^ and the precipitant-related Raman band at 980 cm^-1^ were observed for the HCP spiking solution. As the HCP spiking solution was prepared by recovering the precipitation supernatant and subsequent dialysis, it contained both HCPs and host cell nucleic acids, which was further underlined by a260/A280 ratio of 1.88. The AMS contribution was caused by the residual amounts of AMS after buffer exchange. As the peak profiles of the two spiking solutions in the protein fingerprint region differed slightly from one another and the amide bands are commonly used Raman markers for higher-order structures (Socrates, 2004), the ratio of selected amide bands are presented in Figures 5B–C for selected batch and fed-batch experiments, respectively. The data indicated a shift in the ratio of 1341 and 1660 cm^-1^ over the course of a precipitation experiment depending on the initial conditions of the respective experiment. Exemplarily, the VLP-spike experiments (B6, B7, F2) exhibited slightly lower ratios than the experiments with similar lysate compositions (B4, F1, F3). To compare the intensity decrease observed for the nucleic acid and protein-related bands, the ratio of 781 and 1341 cm^-1^ is shown for batch and fed-batch experiments over increasing AMS content in Figures 5E–F. The curves show a sigmoidal increase indicating a stronger decrease of protein-related Raman bands than observed for the bands associated with nucleic acids. A comparable trend was observed in the A260/A280 ratio for supernatant samples of the batch experiments as presented in Figure 5D. In summary, the differences in protein-associated Raman bands of the two spiking solutions underline structural differences between VLPs and HCPs. Selected band ratios could be used to track these spectral changes over the course of the precipitation, suggesting the selective precipitation of specific molecular species, namely the VLP.

### 3.4 Background correction removes interferences

**FIGURE 6:**
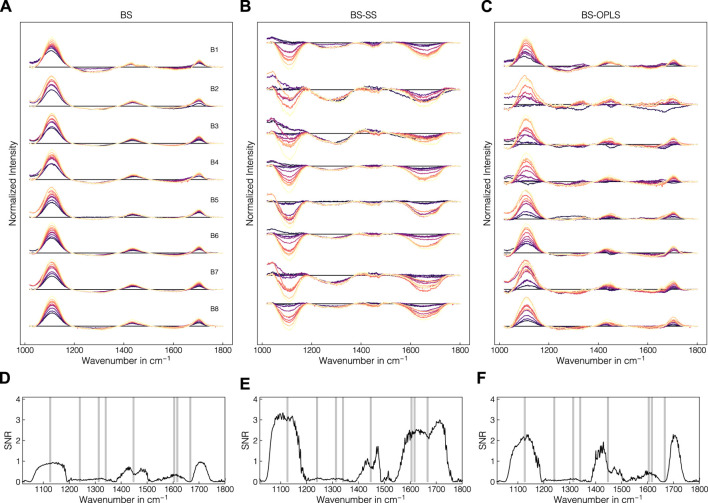
Comparison of the effects of preprocessing operations on Raman spectra in precipitate samples for the wavenumber region 1020–1800 cm^-1^. In **(A–C)**, difference spectra for all batch experiments B1-B8 are shown after turbidity and baseline correction, with incorporated SS-background correction, or with incorporated OPLS-background correction, respectively. The spectra are colored according to the AMS concentration with brighter colors denoting higher concentrations. In **(D–F)**, the SNR for the respective preprocessing operations are shown. Gray shaded areas indicate protein-related Raman regions according to literature (Maiti et al., 2004; Rygula et al., 2013).

The batch experiment data were used to evaluate the suitability of preprocessing pipelines with regard to their universal applicability to all experiments, and their capacity for removing background effects such as buffer and precipitant interferences. Figures 6A–C present the difference spectra after turbidity and baseline correction, incorporated SS-background correction or incorporated OPLS-background correction, respectively, for the precipitate samples of all batch experiments B1-B8. In Figures 6D–F, the SNR is presented to quantify the effect of the baseline and background correction methods on the correlation with the precipitated amount of VLP. The turbidity- and baseline-corrected spectra showed evolving negative bands between 1200 and 1400 cm^-1^ and 1500 and 1700 cm^-1^. These negative bands exhibited SNR values below 0.5. The Raman bands between 1020 and 1200 cm^-1^ and between 1700 and 1720 cm^-1^ showed a clear trend with increasing levels of AMS. The SNR confirmed this observation with the highest values close to 1 for these intervals. Similar trends was observed between 1400 and 1500 cm^-1^ with SNR values up to 0.9. By subtracting the reference spectra comprising solely buffers and precipitants, an additional negative band between 1020 and 1200 cm^-1^ was unveiled and the peak around 1700 cm^-1^ was removed (cf. Figures 6A, B), while the wavenumber region between 1200 and 1400 cm^-1^ remained largely unaffected. The SNR confirmed these observations and showed a strong correlation of the bands between 1020 and 1200 cm^-1^ and 1550 and 1750 cm^-1^, where protein-related Raman bands are located. Bands between 1400 and 1500 cm^-1^ also showed elevated correlation according to SNR compared to sole baseline correction. By incorporating an OPLS-based background correction, the spectra mostly retained positive bands between 1020 and 1200 cm^-1^ and the peaks around 1450 and 1700 cm^-1^ (cf. Figure 6C). However, the SNR revealed that the correlation increase in the range from 1020 to 1200 cm^-1^ and from 1550 to 1750 cm^-1^ is lower than for SS-background correction. Almost no correlation increase was observed near 1600 cm^-1^. Despite the visual differences between the SS- and OPLS-background correction, the SNR assessment showed a similar profile with correlations between 1020 and 1200 cm^-1^, near 1450 cm^-1^ and near 1700 cm^-1^. An analogous illustration of difference spectra and corresponding SNR for the supernatant samples can be found in the Supplementary (Supplementary Figure S1), where comparable but substantially less pronounced trends were observed.

### 3.5 Systematic pipeline optimization improves model accuracy

**FIGURE 7:**
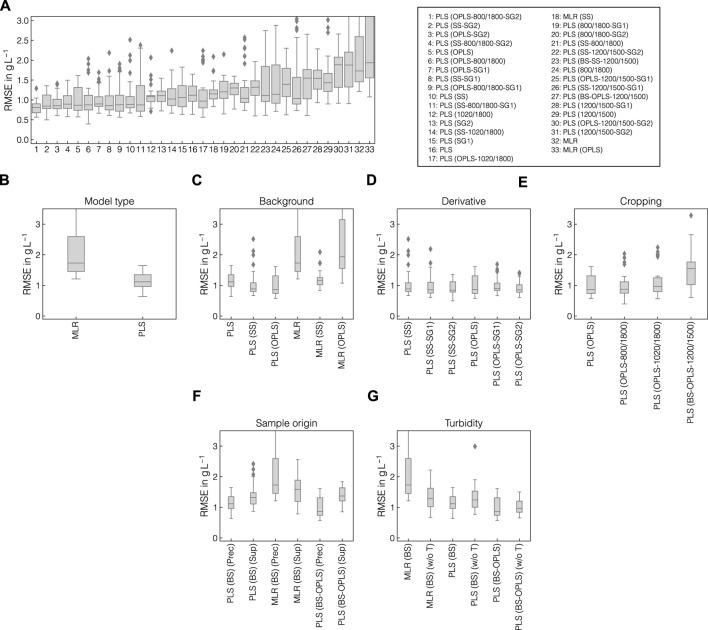
Comparison of preprocessing pipelines and model types. The distributions of RMSE of the outer cross-validation are presented and ranked by the mean of the RMSEs for all tested model configurations **(A)**. All tested model configurations comprised turbidity correction, baseline correction, and difference spectra. The solid lines within the boxes represent the median of the obtained performances. Outliers were characterized by errors surpassing 1.5 times the interquartile range and are symbolized by diamonds. RMSEs of selected model configurations are illustrated separately, namely for comparison of model types **(B)**, background correction methods **(C)**, SGF derivative **(D)**, spectral cropping **(E)**, sample origin **(F)**, and models with and without incorporated turbidity correction **(G)**.

Upon retrieving information about correlative structures in the collected Raman spectra, the data were used to build multivariate regression models taking the Raman spectra as inputs and predicting the apparent precipitated VLP concentration. Multiple combinations of preprocessing operations and MLR or PLS models were screened with the aim to find the optimal pipeline configuration with respect to model accuracy and robustness. While a strong focus lied on evaluating previously assessed operations such as SS and OPLS-based background correction, their combinations with cropping and derivative filters were also evaluated in this Section. In Figure 7A, the distributions of RMSE of the held-out test sets are shown for all tested model and pipeline configurations, sorted by the mean of the RMSEs. Figures 7B–G additionally show the direct comparison of selected model configurations and aim to emphasize the effect of individual changes to the model and preprocessing pipeline on model performance. While the best models achieved RMSE values of approx. 0.8 g L^-1^ on average, the errors increased to up to approx. 2.0 g L^-1^ on average. Model configurations with higher average errors also showed larger variance. Among the four best performing model configurations (cf. Figure 7A), all pipelines included SS or OPLS-background correction, second order SGFs and PLS regression models. While the best and the model ranking 4th used cropping to 800–1800 cm^-1^, the remaining used the full wavenumber range. The PLS model applied to turbidity- and baseline-corrected difference spectra achieved better performance than MLR (cf. Figure 7B). In general, PLS models benefited from using background correction method with the lowest average RMSE and variance being achieved when applying the OPLS method (cf. Figure 7C). For both SS- and OPLS-corrected data, increasing the order of derivative from 0 to 2 marginally improved the median RMSE and reduced variance (cf. Figure 7D). Cropping the Raman spectra generally showed adverse effects on model accuracy using OPLS-corrected data (cf. Figure 7E). As pointed out above, this was not true for all pipeline configurations but may be pointed out as a general tendency in this case study. To validate that the identified pipelines can not only be applied to Raman spectra recorded from the precipitate-containing samples, but also to the particulate-free supernatant samples, separate models were calibrated on the supernatant Raman spectra as shown in Figure 7F. While the PLS and MLR models achieved similar accuracy after turbidity and baseline correction in precipitate and supernatant samples, OPLS-background-corrected models showed considerably increased RMSE on average in the supernatant samples. In Figure 7G, separate models were calibrated without turbidity correction to verify that model performance benefit from the incorporated turbidity correction. Although MLR and PLS models achieved comparable performance when applied to solely baseline-corrected difference spectra, MLR performed worse than PLS models when combined with turbidity correction. All tested model and pipeline configurations without turbidity correction can be found in Supplementary Figure S2.

### 3.6 Model pipeline captures precipitation trends

**FIGURE 8:**
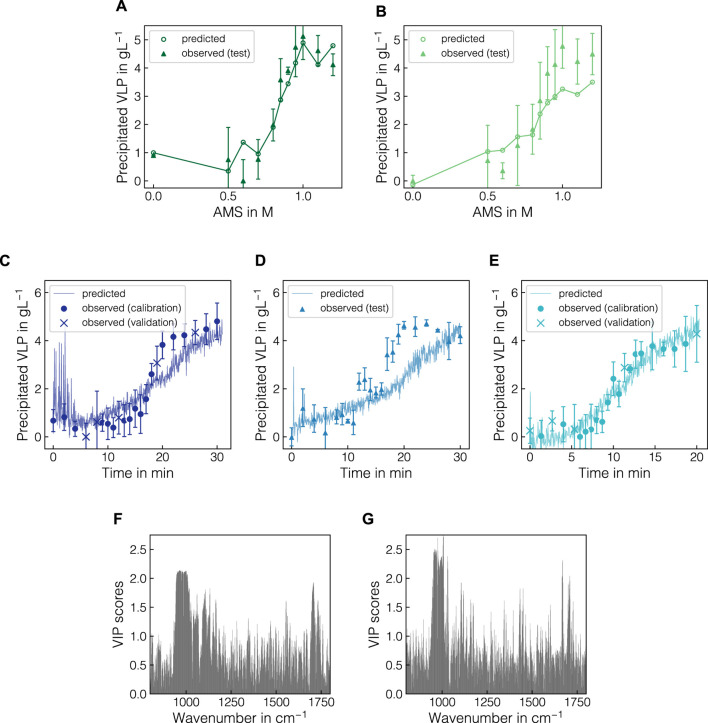
PLS model predictions with regard to precipitated VLP for batch and fed-batch experiments and the corresponding VIP scores for both models. For the batch experiments B4 **(A)** and B6 **(B)**, the predicted and observed precipitated VLP concentrations are shown over increasing AMS concentrations. For visual purposes, solid lines are shown to linearly connect the PLS model predictions. For the fed-batch experiments F1-F3 **(C–E)**, the observed apparent VLP concentrations are shown as symbols with their respective assignment to calibration, validation and test data. Model predictions from the real-time data after baseline correction and difference spectra are presented as solid lines. The VIP scores are illustrated for both batch **(F)** and fed-batch **(G)** models in the wavenumber region 800–1800 cm^-1^.

The previously identified best model and preprocessing pipeline PLS(BS-OPLS-800/1800-SG2) was finally evaluated on a representative split of batch data and further transferred to the fed-batch data. Figure 8 presents PLS model predictions for the batch test set (B4, B6) and fed-batch experiments F1-F3 as well as VIP scores for both calibrated models. Despite having different VLP to impurity ratios in the starting material (cf. Table  1), the PLS model predictions for batch experiments B4 and B6 aligned well with the experimental data (cf. Figures 8A, B) with an *R* ^2^ of 0.83. Error metrics for calibration, cross-validation and test sets are presented in Supplementary Figure S3. Alternatively, PLS model predictions for all calibration runs are shown over AMS content in Supplementary Figure S4. While for B4, the sigmoidal shape of the precipitation curve was represented in the model predictions, the model predicted an early onset of the VLP precipitation for B6 and saturate at lower final concentration of approx. 3.5 g L^-1^, effectively smoothing out the sigmoidal shape. For the calibration experiments (cf. Supplementary Figure S4), the sigmoidal shape and onset of the precipitation were predicted accurately while predictions for the initial plateau differed from the observations. Most of the predictions, however, lay within the range of one standard deviation of the observed data points.

In Figures 8C–E, the predictions of the calibrated PLS model are shown for fed-batch experiments F1, F2 and F3 as symbols with their respective assignment to calibration, validation and test sets. Supplementary Figure S5 presents error metrics for all data sets. The real-time data used for prediction were not averaged and hence the model predictions were scattered around the observed data points and were available in between reference measurements. While the trajectories of the expected precipitation curves were well represented in the predictions for the training experiments F1 and F3, the trajectory for F2 was underestimated in the range from 15 to 25 min which corresponded to approx. 0.5–1 M AMS. Using the averaged Raman spectra, this yielded an *R* ^2^ of 0.64 (cf. Supplementary Figure S5). For F1, the predictions for the spectra during the first 3 min strongly scattered and deviated from the expected values. Similar spikes for individual spectra were observed in the start-up phase for F2 and F3. In all cases, the effect reduced over the first 1–3 min after which constant scatter can be observed for all experiments until the end of the precipitation process. To evaluate the origin of these scatter, a step-wise visualization of the Raman spectra for all fed-batch experiments is presented in Supplementary Figure S8. The affected Raman spectra show slight distortions to lower intensity in the protein-related and water Raman bands, which are subsequently enhanced through the sequence of preprocessing operations. Towards the end of the process, no plateauing of the predictions based on the Raman data was observed whereas the observed data signals tended to stabilize with individual spikes being present.

Finally, Figures 8F, G present the VIP-based feature importance for the PLS models based on batch and fed-batch data, respectively, in the wavenumber range between 800 and 1800 cm^-1^. Both models relied on multiple wavenumber regions as indicated by elevated VIP scores. Most prominently, the interval between 950 and 1000 cm^-1^ originating from the sulfate ions contributed to the model predictions. Additionally, local maxima appeared at wavenumber intervals of 800–900, 1050–1150, 1400–1500 and 1550–1650 cm^-1^ for both the batch and the fed-batch models, mainly encoding protein-related information.

PLS models were further used to quantify the AMS concentration in batch and fed-batch using the same methodology as laid out for the precipitated VLP concentration. In the case of AMS the preprocessing only included turbidity and baseline correction and the formation of difference spectra. The predictions for the batch and fed-batch data are presented in Supplementary Figures S6, S7, respectively. For both data sets, the models showed almost perfect alignment for the test sets with *R* ^2^ of 0.98 and 0.97 over the concentration range from 0 to 1.2 M. For the fed-batch runs deviations from the expected values were observed above AMS concentrations of 1.2 M.

## 4 Discussion

### 4.1 Effects of data diversification

Data diversification is crucial for generating representative data sets for developing chemometric sensors suitable for PAT in biomanufacturing. The precipitation experiments were designed to mimic multiple sources of experimental variance which may arise in process development studies or manufacturing campaigns. Data were diversified using different lysate batches and conditioning of the lysates by dilution, salt addition, or spiking with purified VLP or HCP solutions. Similar strategies for data diversification were employed in the literature by spiking purified monoclonal antibodies with mock solutions to mimic complex feedstocks (Großhans et al., 2019), spiking with nutrients during cell culture (Santos et al., 2018), spiking with cells during inoculum maturation (Guardalini et al., 2023b) or mixing available samples (Wang et al., 2023). In general, diversification strategies were reported to improve model robustness and interpretability (Santos et al., 2018) or enlarge the experimental data set (Wang et al., 2023). Alternative strategies may include synthetic enlargement of the available data sets by data augmentation (Schiemer et al., 2024) or the collection of data from multiple products, formulation components and sensors (Wei et al., 2022) for the diversification of experimental data sets.

In essence, the diversification strategy in this study yielded versatile precipitation experiments covering different precipitation trajectories, varying levels of total concentration of precipitated VLP and impurity content. However, the UV absorbance data exhibited high variance for the triplicate measurements which was enhanced by the conversion to the apparent precipitated VLP concentration, resulting in difficulties in determining the precipitation trajectories. Particularly, samples in the plateau region of the precipitation curves showed unexpected fluctuations. The variance between replicates or individual data points within one experiments, i.e. variable onsets of the precipitation or the initial and final plateau concentration, may be attributed to the precipitation procedure, sampling procedure and sample handling. Since the batch experiments involved the manual addition of the precipitant as well as sampling of the precipitate-containing solution, the heterogeneity of the precipitate solution may introduce analytical variance between data points. Moreover, inadequate mixing during precipitant addition or during the subsequent incubation time could also contribute to variance within a batch experiment. Variance between replicates and individual data points were less pronounced for fed-batch than batch experiments, which can be attributed to the inherent continuity of the fed-batch approach as well as effective mixing in the reservoir, thus, supporting uniform precipitate formation while avoiding co-precipitation of other species (Pons Royo et al., 2022).

Eventually, the observed variances led to the employment of a pretreatment strategy based on experiment-based curve fitting using the Boltzmann function. This strategy assumed the selective precipitation of VLPs which has been shown for AMS concentrations below approx. 1.3 M (Wegner and Hubbuch, 2022). The pretreatment strategy was shown to effectively reduce single-point variation by removing outliers and resolving differences between the precipitation experiments. Treatment of the reference data is uncommon in chemometrics but certainly improved the quality of the data in this study and enabled more robust model calibration (data not shown).

In summary, it is crucial to manage data variance within individual experiments, e.g. by increasing the number of replicates or incorporating a physically-motivated pretreatment, while highly diversifying data across experiments, as the obtained batch and fed-batch data sets served as basis for the development of a chemometric modeling pipeline for the quantification of precipitated VLPs.

### 4.2 Effects of preprocessing operations on Raman data

The Raman spectra are affected by the composition of the lysate, the addition of the precipitant and the formation of precipitates in solution causing multiple independent changes in the Raman spectra to occur simultaneously. While the addition of the precipitant induced the emergence of the sulfate and ammonium-associated Raman bands, the formation of precipitates reduced the overall intensity due to turbidity of the solution. Turbidity is commonly observed in particulate-containing separation processes such as crystallization (Moreno et al., 2000; Grön et al., 2003; Harner et al., 2009; Liu et al., 2014) or precipitation (Meyer-Kirschner et al., 2016; Zelger et al., 2016). While Zelger et al. (2016) directly used the turbidity measurements for monitoring the precipitation progress, Meyer-Kirschner et al. (2016) demonstrated that a simple correction using the OH-stretching Raman band is feasible. Since regulatory agencies (FDA, 2015) recommend rigorous qualification of PAT sensors, the authors consider the use of Raman spectroscopy in combination with turbidity correction a viable PAT sensor for monitoring protein precipitation processes. Here, normalization at the OH-stretching Raman band reliably compensated for turbidity in the raw spectra. However, it was also pointed out that the turbidity correction adversely affects other wavenumber regions in the herein recorded, baseline-corrected spectra such as the sapphire band at 750 cm^-1^. This may be connected to the non-linear correlation between turbidity and Raman intensity decrease (Sinfield and Monwuba, 2014).

After turbidity and baseline correction, the variation in the protein-associated Raman bands was comparable to what has been observed for the supernatant spectra while the inexplicable decrease in nucleic acid-associated bands was eliminated. This supports the assumption of selectivity of the precipitation process for VLPs. A direct quantification of the amount of HCPs was not possible due to the unavailability of quantitative reference measurements in this study. The spectral data of the HCP- and VLP-enriched solutions, however, suggest that the VLPs are structurally different from the HCPs contained in the clarified cell lysate which is supported by different amide band ratios at the start and the end of a precipitation experiment.

While this study was performed using HBcAg VLPs, the applicability of Raman spectroscopy for HBcAg variants or other types of VLPs is expected. To apply the presented workflow to other VLP systems, the generation of new experimental data is strictly necessary. Furthermore, by incorporating additional reference analytics such as HCP-enzyme-linked immunosorbent assay (ELISA) as done in Großhans et al. (2019), the prediction of HCP content via Raman spectroscopic measurements may hence be facilitated. However, the prediction of HCPs using chemometric methods has only rarely been reported (Capito et al., 2015; Chen et al., 2024), most likely due to the diversity of species classified as HCPs (Falkenberg et al., 2019). The same applies to nucleic acids and endotoxins, which might affect VLP precipitation, but their analysis exceed the scope of this study.

To prepare spectral data for multivariate modeling, turbidity, baseline and background correction followed by the difference spectra operation were evaluated for their correlation with the amount of precipitated VLP and their capacity to eliminate interferences in the Raman spectra caused by the addition of precipitant. Baseline correction of turbidity-corrected spectra proved to remove baseline drifts reliably across the entire wavenumber range. As the applied asPLS combines baseline estimation with smoothing of the estimated function while preserving the underlying features of interest (Zhang et al., 2020), baseline drifts caused by both sample compounds and instrumental noise can be addressed.

The SNR-based correlation analysis of the baseline-corrected spectra suggested that next to protein-associated Raman bands, wavenumber regions encoding buffer and precipitant-associated information were also correlated with the amount of precipitated VLP. As the increase in precipitated VLP is expected to follow a sigmoidal curve (Valentic et al., 2022; Wegner and Hubbuch, 2022), the precipitant concentration in the solution and the amount of precipitated VLP is not linearly correlated. Hence, the contributions of the precipitant should be removed from Raman spectra via background correction. The SS-background correction reliably removed the background signal from the Raman spectra, while overcorrections were visible for samples with AMS concentrations above 0.6 M (cf. Figure 3). This is caused by the discrepancy with regards to the turbidity between the precipitate spectra and the reference spectra where the solution remains clear even at the highest AMS concentrations of 1.2 M. The OPLS method as proposed by Trygg and Wold (2002) demonstrated improved correlations by removing the non-correlated systematic variation in the Raman spectra without the need for reference measurements. However, also the OPLS-treated spectra showed residual contributions in the dominant 980 cm^-1^ sulfate band and hence could not fully remove the precipitant-associated effects. As the OPLS method extracts the non-correlated variation with respect to the target variable (Trygg and Wold, 2002), it is likely that the AMS-associated information was partially contained due to its correlation with the amount of precipitated VLP as mentioned before. Alternatively background variations may be modeled using indirect hard modeling (IHM) (Alsmeyer et al., 2004; Kriesten et al., 2008; Meyer-Kirschner et al., 2016). Furthermore, the extended multiplicative signal correction (EMSC) algorithm supports the removal of an interferent spectrum (Martens and Stark, 1991). However, Liland et al. (2016) reported its propensity for overfitting and its implementation has been found ineffective in this study (data not shown).

Finally, all Raman data were treated using the difference spectra operation by subtracting the first Raman spectrum in each experiment. Difference spectra are a common method to emphasize temporal changes in spectroscopic data (Andris et al., 2018) or differences between individual samples (Gautam et al., 2015; Zhou et al., 2015). The difference spectra operation was shown to effectively remove all variations in the initial composition of the studied lysate using different conditioning approaches. This improved comparability between experiments and specificity of the Raman signals for the amount of precipitated VLPs which is crucial for generating robust chemometric models (Santos et al., 2018).

In summary, the preprocessing pipeline comprising turbidity and baseline correction, a background correction technique and difference spectra contributes to the overall quality of the precipitate Raman data, improving model building. The identified preprocessing pipeline may further be transferable to other precipitants such as polyethylene glycol (PEG). In the case of PEG-based systems, it may be more challenging to resolve the differences between the precipitants and protein-related signals due to organic Raman contributions of the PEG polymers (Kuzmin et al., 2020).

### 4.3 Effects of preprocessing pipeline on model performance

Chemometric models based on Raman spectroscopy data often employ multiple preprocessing operations in sequence and require optimization of the associated hyperparameters (Bocklitz et al., 2011; Gerretzen et al., 2015; Brunel et al., 2021). To study the effect of individual preprocessing operations on model performance, multiple pipeline configurations were screened and evaluated using a nested cross-validation approach. The nested cross-validation enabled the comparison of the pipeline configurations, not only with regard to the overall accuracy, but also their robustness when permuting the training and test sets. By further incorporating interval-based cropping of the Raman spectra and the comparison of MLR with PLS models, the understanding for the relevance of specific features in the Raman data could be increased.

In general, PLS models showed considerably better performance and lower variance in cross-validated test sets than MLR models. This may be explained by the high complexity of Raman data and the experimental variance which was observed for the batch data set. The MLR models solely rely on VLP-associated Raman bands and hence require high-quality data. The PLS models may also use other correlative information available in the Raman spectra (Santos et al., 2018; Goldrick et al., 2020) which is not necessarily specific for the precipitation of VLPs such as the turbidity or precipitant markers. Nevertheless, the comparison with MLR as a benchmark is a useful strategy as in less complex cases, the relevant information may be reliably contained in a few individual variables (Hillebrandt et al., 2022).

As anticipated from the SNR analysis, both background correction approaches improved the performance of the PLS models due to the removal of precipitant-related Raman bands. As the AMS contribution dominated the Raman spectra and was recovered in the first latent variables of the PLS models, models without prior background correction may rely too strongly on background information and hence not robustly predict the precipitated amount of VLP. Recent studies with biopharmaceuticals often rely on standardization, derivative filtering or baseline correction (Santos et al., 2018; Wei et al., 2022; Rolinger et al., 2023; Wang et al., 2023). Others performed background corrections by subtracting blank spectra but did not further investigate the importance of the respective features on model predictions (Großhans et al., 2019; Rolinger et al., 2021; Weber and Hubbuch, 2021). Alternatively, Wang et al. (2023) performed blank chromatography runs with the elution buffer system and appended the recorded data to the calibration data set.

To assess whether the focus on spectral regions of interest improves model predictions, multiple cropping intervals were explored within this study. Cropping the spectra to 800–1800 cm^-1^ generally improved model performance, whereas further cropping decreased the overall accuracy of the PLS models. While most of spectral information is still contained in the 800–1800 cm^-1^ region, narrowing the wavenumber region to 1200–1500 solely focused on the protein-related information (Maiti et al., 2004; Rygula et al., 2013). The narrow bandwidth was not suitable to reliably predict precipitated VLP concentrations and notably broadened the distribution of RMSE while increasing the mean error by over 50%. This may be explained by the strong variances in the sensor data between experiments leading to non-linear correlations between the spectral data and the amount of precipitated VLP. When using B4 and B6 as the hold-out test set, a RMSE of 0.74  gL^-1^ was reached, supporting the VLP-associated information being stored in the respective wavenumber regions. Because the stated RMSE lies in the center of the RMSE distribution of the held-out test sets (cf. Figure  7), the data split is considered representative.

The optimal performance in the permuted batch data was found for the PLS model for the combination of turbidity, baseline, and OPLS-based background corrections with cropping to 800–1800 cm^-1^ and second derivative filtering. When applied to the representative splits of the batch and fed-batch data, the identified model pipeline recovered the observed trends in the experimental data but lacked to resolve the correct sigmoidal shape for experiments B7 and F2. While the pipeline proved robust against background variations induced by lysate conditioning and spiking, the sigmoidal shape of the VLP precipitation trajectories and the height of the final plateau were not exactly met. Due to the overlaying effects occurring in the Raman spectra, the insufficient removal of the background contributions by the OPLS method and the high single-point variance in the reference data, the relationship between the VLP-related Raman bands and the target variable is considered to be non-linear. By inspecting the importance of different wavenumber regions through VIP, the model’s reliance on residual precipitant information and the VLP-associated bands became apparent. Moreover, the VIP scores suggested a strong influence of noise in the spectra on the model predictions as no Raman band is clearly recovered. Despite being robust to background variations, the preprocessing pipeline may also be influenced by unforeseen changes in the Raman spectra due to equipment failure or other experimental factors. In the case of the fed-batch experiments, the scattered predictions for the spectra during the first 3 min are likely due to a combination of factors. These include experimentally introduced air bubbles or large flocculates, which can distort the Raman signal, and overcorrections from preprocessing operations that may perform differently when the Raman spectra show unforeseen changes. Local concentration gradients due to possible heterogeneities in precipitant concentration could also cause scattered predictions, but there is no evidence for this in the AMS-related Raman bands.

To further study the effect of noise on the model, perturbation studies (Wang et al., 2023) could be employed or the distribution of PLS weights could be assessed (Cui and Fearn, 2018). To effectively reduce the noise in the model, a larger data set preferably from fed-batch experiments would be required. Eventually, due to the non-linear relationship of the Raman spectra and the VLP concentration, non-linear regression models should be evaluated such as kernel-based methods (Thissen et al., 2004; Barman et al., 2010; Zavala-Ortiz et al., 2020; Schiemer et al., 2023) or neural networks (Cui and Fearn, 2018; Wang et al., 2023; Schiemer et al., 2024).

## 5 Conclusion and outlook

In conclusion, we present a Raman spectroscopy-based PAT for real-time monitoring of VLP precipitation from clarified *E. coli*-derived lysate as well as the precipitant concentration. The generated precipitation data provide a challenging data set with varying precipitation dynamics and backgrounds which were induced by spiking and are desirable for robust PAT sensor development. High experimental variance is successfully managed by employing a pretreatment approach for the UV absorbance data. The preprocessing pipeline including turbidity, baseline and OPLS-based background correction, difference spectra, cropping and derivative filtering is identified as optimal for removing all variations in the initial composition of the studied lysates as well as most interferences caused by precipitates and precipitant in solution. The final PLS models recover the observed trends in the batch and fed-batch data but lack to resolve fine differences between experiments owing to the non-linear relationship between spectral data and the precipitated VLP concentration. Additionally, the Raman data reveal structural differences between VLPs and HCPs and qualitatively support the selective precipitation of VLPs while nucleic acids and HCPs remain in solution. Overall, the developed preprocessing pipeline provides a foundation for integrating Raman spectroscopy as a PAT sensor for monitoring particulate-containing bioprocesses and bears the potential to be applied to other phase behavior-dependent processes for protein purification.

## Data Availability

The raw data supporting the conclusion of this article will be made available by the authors, without undue reservation.
