# Supplementary material for: Raman-based PAT for VLP precipitation: systematic data diversification and preprocessing pipeline identification
Source: Front Bioeng Biotechnol. 2024 May 31;12:1399938. doi: 10.3389/fbioe.2024.1399938 (PMC11177211; doi:10.3389/fbioe.2024.1399938)
Supplement: Supplementary file 1 [file DataSheet1.PDF]

Supplementary Material

1 SUPPLEMENTARY DATA

1.1 Buffers, solutions, and spiking materials

If not otherwise stated, chemicals (Merck KGaA, Darmstadt, DE) and ultrapure water (PURELAB Ultra, ELGA LabWater, Lane End, High Wycombe, UK) were used for buffer and stock solution preparation. Buffers were pH-adjusted with 32 % HCl using a SenTix62 pH electrode (WTW, Weilheim, DE) coupled to a HI 3220 pH meter (Hanna Instruments, Woonsocket, US). Buffers and stock solutions were filtered through a 0.2 µm pore-size cellulose acetate filter (VWR International, Radnor, US). For all experiments, stock solutions used for polysorbate adjustment and precipitation experiments were 10% (v/v) polysorbate 20 and 4 M ammonium sulfate (AMS), respectively. Lysis or high-salt lysis buffers consisted of 50 mM Tris, 100 mM or 1500 mM NaCl, 1 mM EDTA (AppliChem GmbH, Darmstadt, DE) at pH 8.0, respectively.

For the virus-like particle (VLP)-enriched spiking material, clarified lysate was further purified by precipitation and re-dissolution, diafiltration, and chromatography as described by Hillebrandt et al. (2021). The purified VLPs were further dialyzed into lysis buffer overnight using 10 kDa MWCO Slide-A-Lyzer G2 cassettes (Thermo Fisher Scientific Inc., Waltham, US) and concentrated by ultrafiltration with Vivaspin 20 centrifugal filters (Sartorius Stedim Biotech GmbH, Göttingen, DE) in a 5810R centrifuge (Eppendorf, Hamburg, DE). VLP concentration was determined using a NanoDrop 2000c UV/Vis spectrometer (Thermo Fisher Scientific Inc.) and the theoretical extinction coefficient at 280 nm of 1.764 $L\,g^{-1}\,cm^{-1}$ as provided by the ProtParam tool (Gasteiger et al., 2005). VLP-enriched spiking material concentrations were 6.95 $g\,L^{-1}$ for batch precipitation experiment B5 and 3.85 $g\,L^{-1}$ for batch and fed-batch precipitation experiments B6 and F2, respectively. For both VLP-enriched spiking materials, a A260/A280 ratio of 0.63 indicated almost pure protein.

For the host-cell protein (HCP)-enriched spiking material, clarified lysate was adjusted to 0.25% (v/v) polysorbate 20 and precipitation was conducted at 1.1 M AMS for 30 min under stirred conditions. Precipitate solution was centrifuged at 12000 G and 4 °C for 30 min. Supernatant was collected and a similar procedure of dialysis and concentration was performed as for the VLP-enriched spiking material, but using 2 kDa MWCO devices. HCP-enriched spiking material characteristics were a A280 value of 57.3 mAU and a A260/A280 ratio of 1.88, both indicating host-cell nucleic acids in the spike solution.

2 SUPPLEMENTARY TABLES AND FIGURES

2.1 Figures

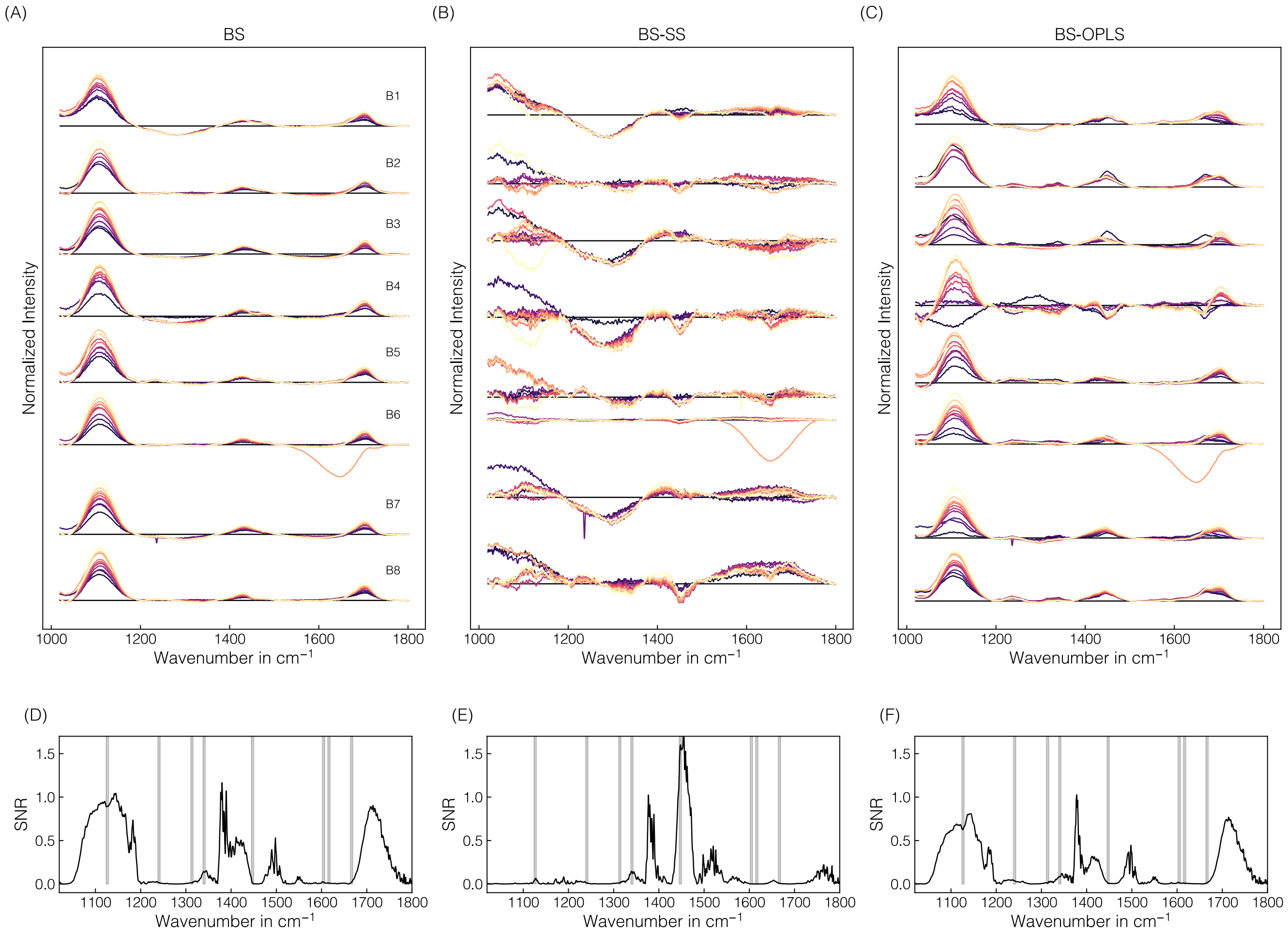

Figure S1. Comparison of the effects of preprocessing operations on Raman spectra in supernatant samples for the wavenumber region 1020-1800 cm^{-1}. In **(A)-(C)**, difference spectra are shown for all batch experiments B1-B8 after turbidity and baseline correction, with incorporated scaled substraction (SS)-background correction, or with incorporated orthogonal projection to latent structures (OPLS)-background correction, respectively. The spectra are colored according to the AMS concentration with brighter colors denote higher concentrations. In **(D)-(F)**, the corresponding signal-to-noise ratio (SNR) are shown. Gray shaded areas indicate protein-related Raman regions according to literature (Maiti et al., 2004; Rygula et al., 2013).

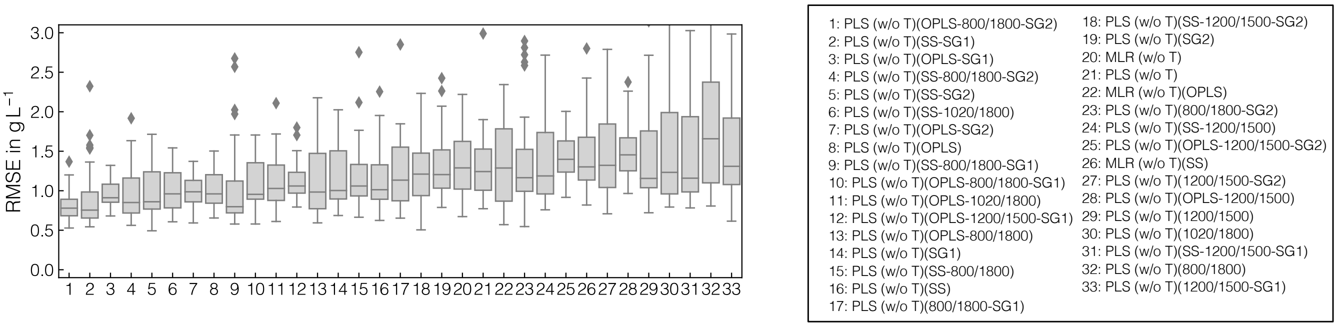

Figure S2. Comparison of model types and preprocessing pipelines without turbidity correction. The distributions of root mean squared error (RMSE) of the outer cross-validation are presented and ranked by the mean of the RMSEs for all tested model configurations. All tested model configurations comprised baseline correction and difference spectra. The solid lines within the boxes represent the median of the obtained performances.

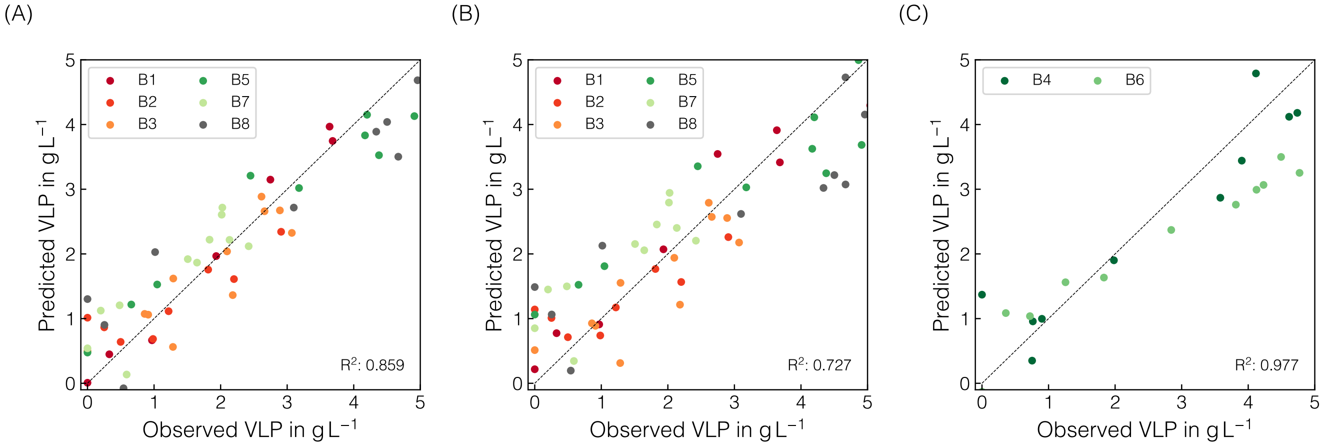

Figure S3. Partial least squares (PLS) model predictions with regard to precipitated VLP concentration for batch experiments. The predicted and observed VLP concentrations for calibration **(A)**, cross-validation **(B)** and test sets **(C)** are plotted against each other with the tie line indicating the optimal result.

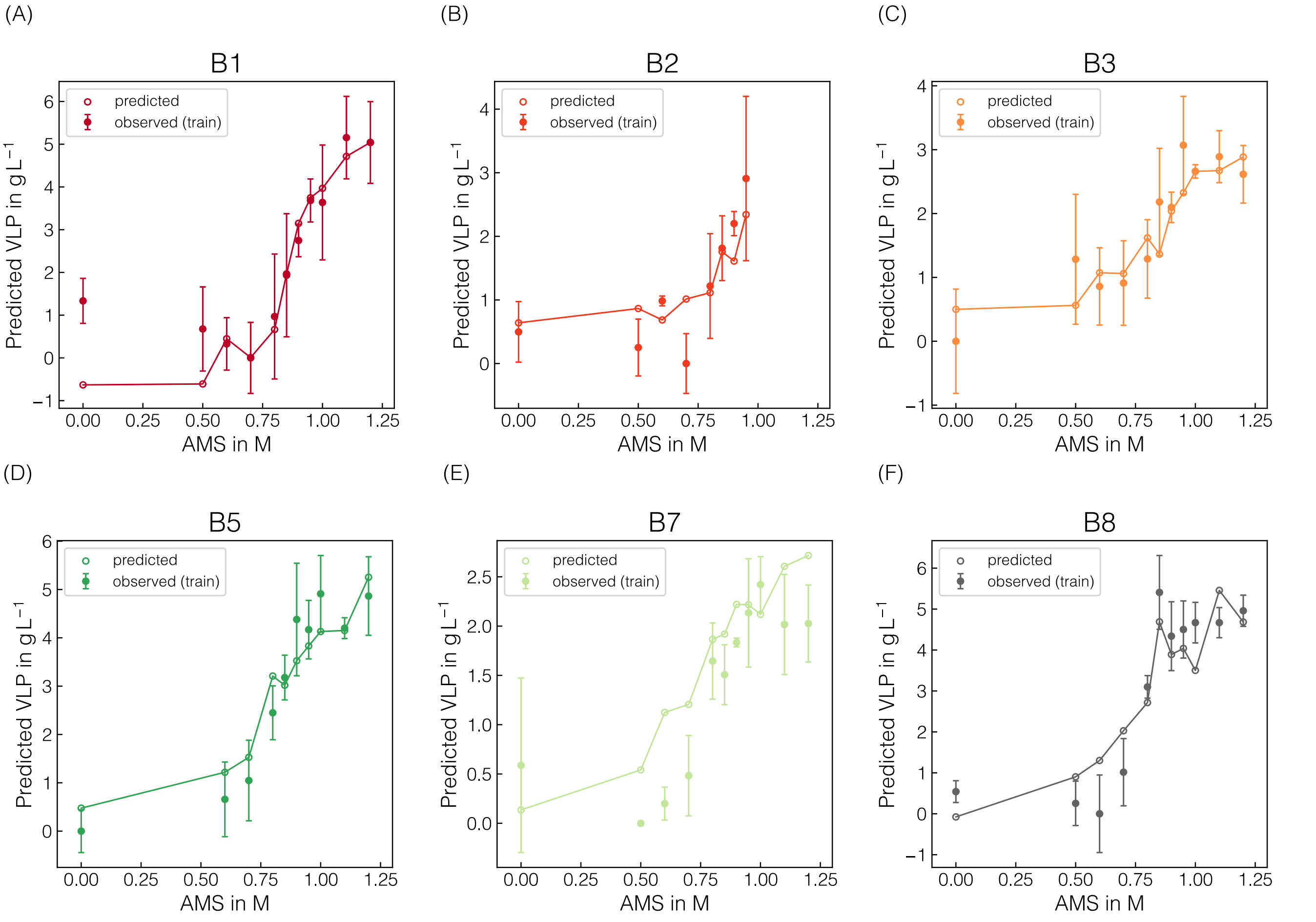

Figure S4. PLS model predictions with regard to the precipitated VLP concentration for batch experiments over AMS concentrations. Batch experiments assigned to the training set (B1, B2, B3, B5, B7, B8) are shown in **(A)**-**(F)**, respectively. The predicted and observed VLP concentrations for all calibration experiments are displayed as empty and full circles, respectively. For visual purposes, solid are shown to linearly connect the PLS model predictions. For batch experiment B2, the data points above 0.95 M were excluded due to defective Raman spectra.

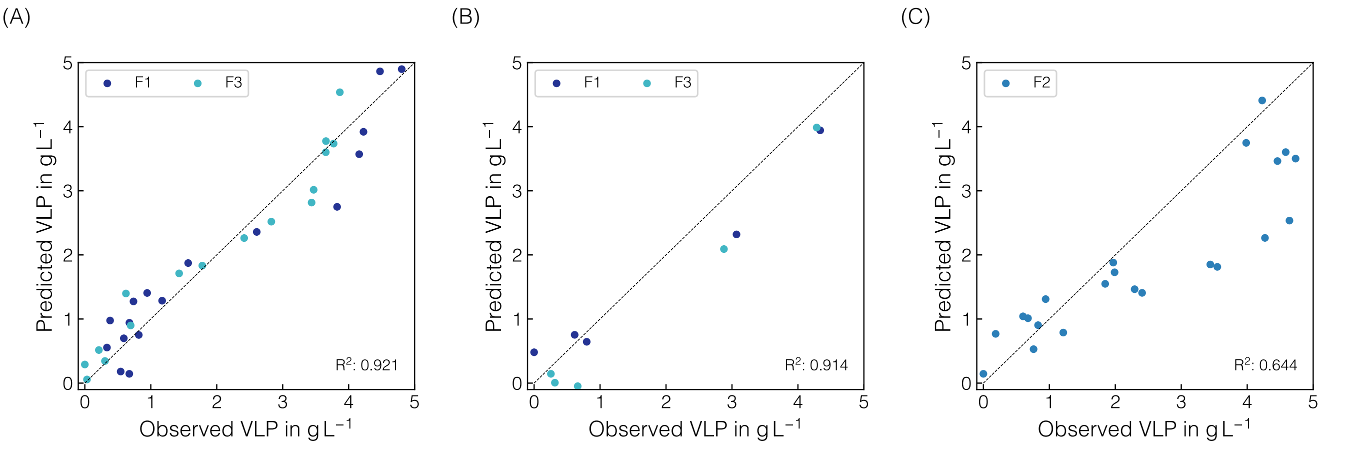

Figure S5. PLS model predictions with regard to precipitated VLP concentration for fed-batch experiments. The predicted and observed VLP concentrations for calibration **(A)**, validation **(B)** and test sets **(C)** are plotted against each other with the tie line indicating the optimal result.

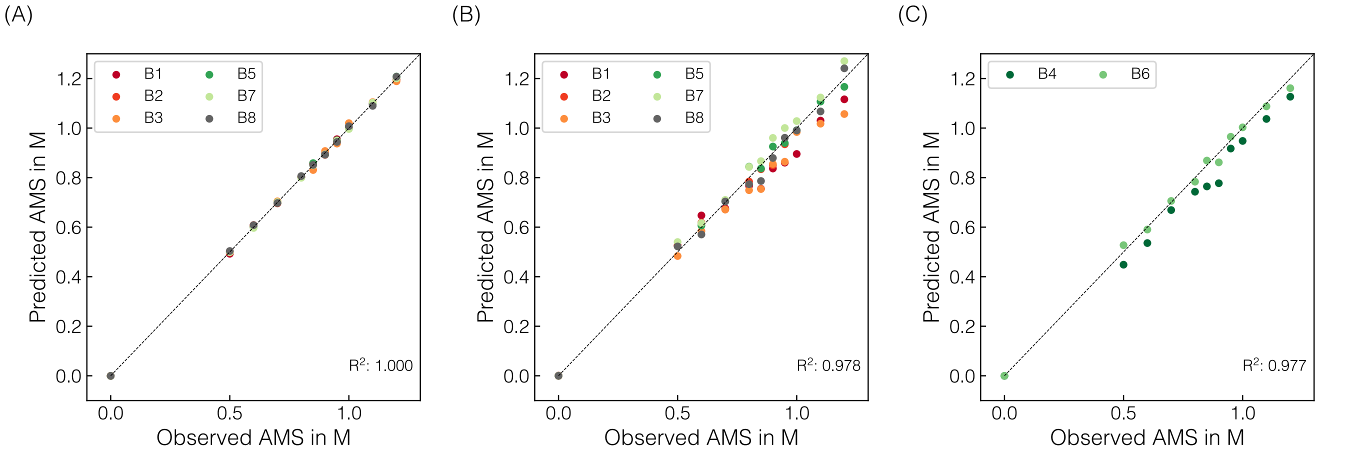

Figure S6. PLS model predictions with regard to AMS concentration for batch experiments. The predicted and observed AMS concentrations for training **(A)**, cross-validation **(B)** and test sets **(C)** are plotted against each other with the tie line indicating the optimal result.

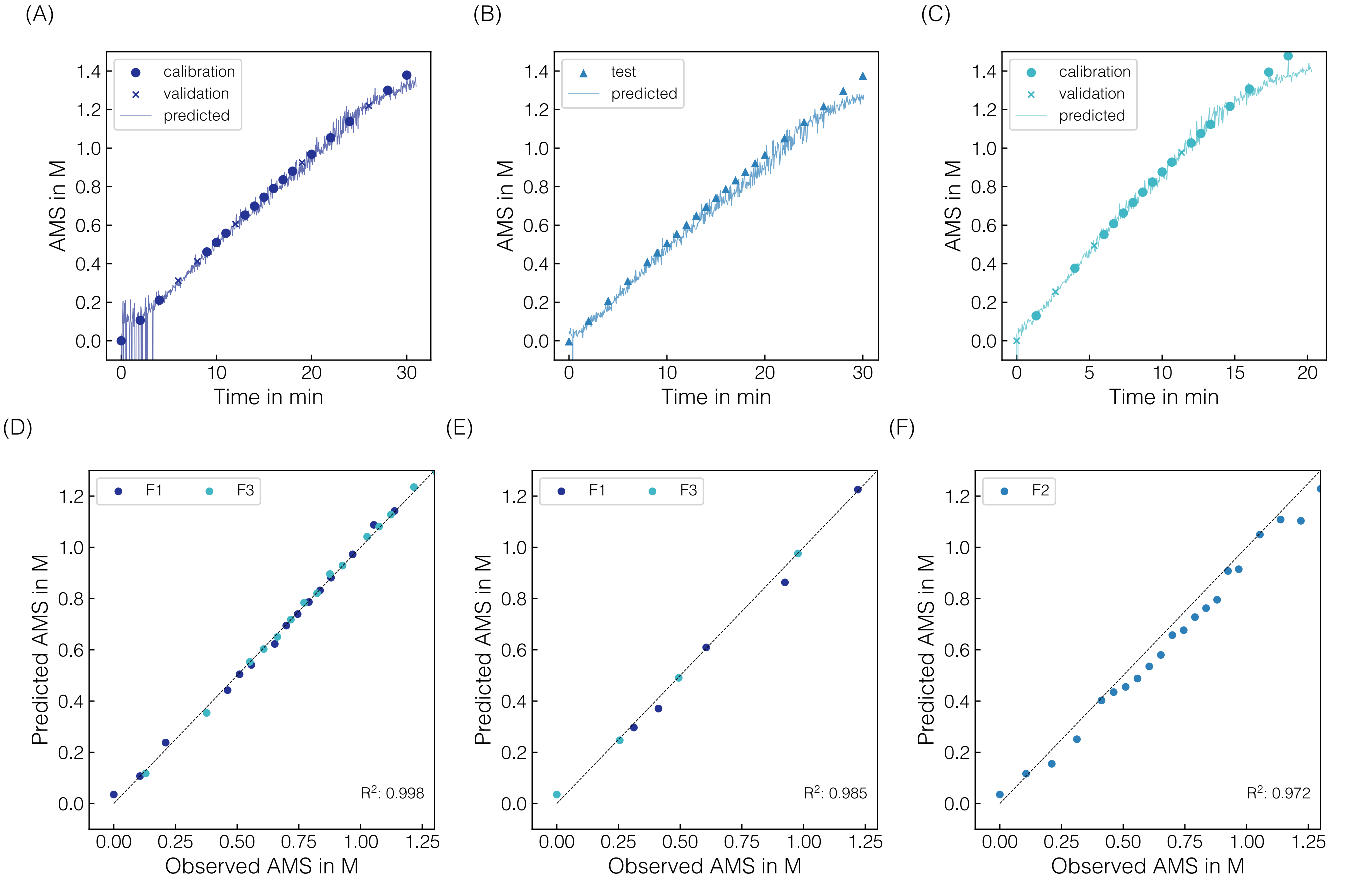

Figure S7. PLS model predictions with regard to AMS concentration for fed-batch experiments. In **(A)-(C)**, the timely predictions obtained from real-time Raman spectra are shown as solid lines and observed AMS concentrations are shown as scattered points. The data points belonging to the calibration, validation and test sets are marked with circles, crosses and triangles, respectively. The PLS predictions for the calibration, validation and test sets are plotted against the observed AMS concentrations in **(D)-(F)**, respectively.

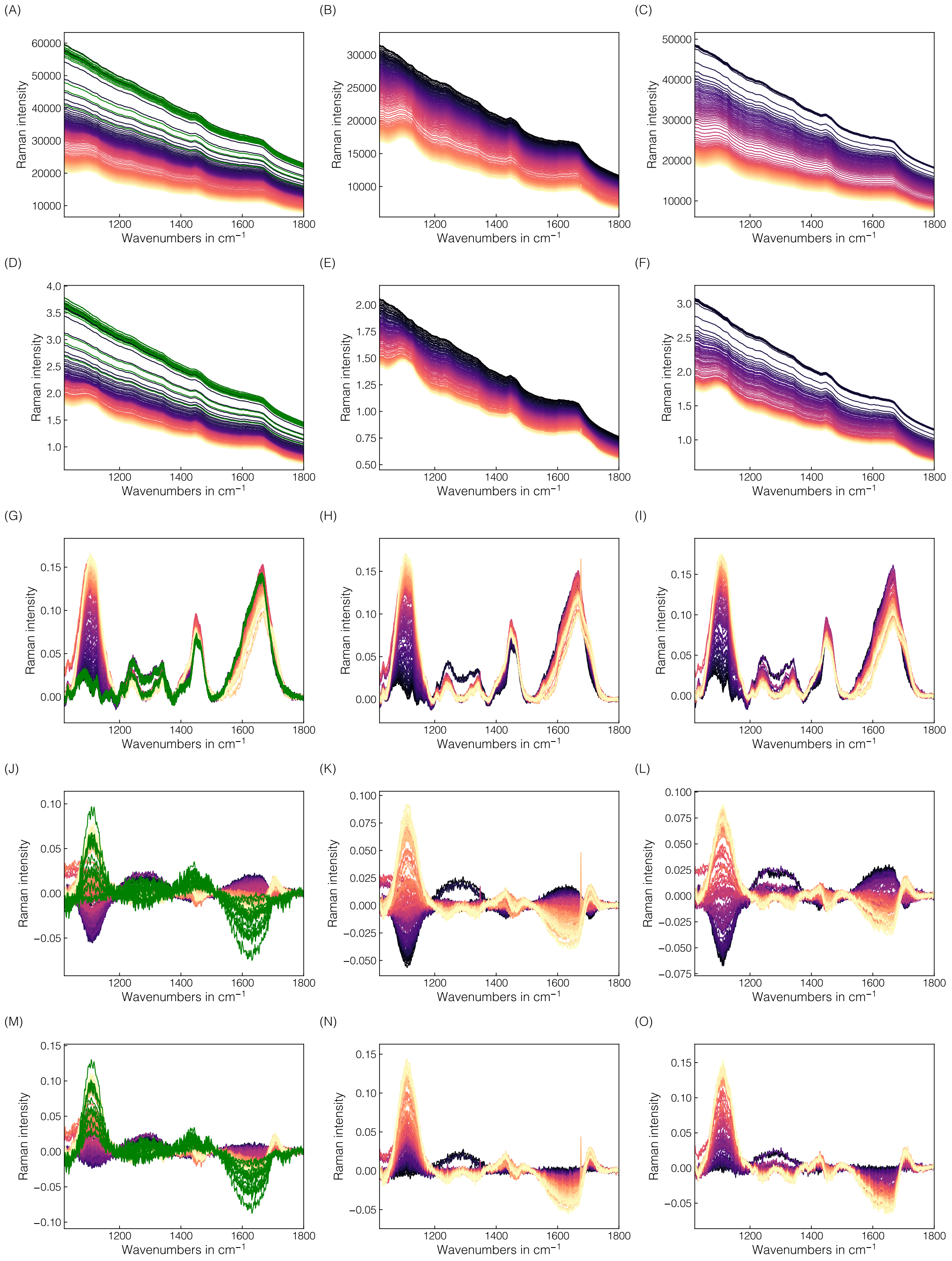

Figure S8. Visualization of preprocessing operations for fed-batch experiments F1-F3 from left to right. The individual rows depict the Raman spectra at different stages of the preprocessing pipeline: Raw **(A)-(C)**, turbidity correction **(D)-(F)**, baseline **(G)-(I)**, OPLS background **(J)-(L)** and difference spectra **(M)-(O)**. For visual purposes, only every 5th spectra from each fed-batch experiment is shown. The Raman spectra affected by scattering in the PLS predictions are marked in green. The Raman spectra are colored according to the theoretical AMS concentration in the system with brighter colors denoting higher concentrations.

REFERENCES

Gasteiger, E., Hoogland, C., Gattiker, A., Duvaud, S., Wilkins, M. R., Appel, R. D., et al. (2005). Protein Identification and Analysis Tools on the ExPASy Server. *The Proteomics Protocols Handbook* , 571–607doi:10.1385/1-59259-890-0:571

Hillebrandt, N., Vormittag, P., Dietrich, A., Wegner, C. H., and Hubbuch, J. (2021). Process development for cross-flow diafiltration-based VLP disassembly: A novel high-throughput screening approach. *Biotechnology and Bioengineering* 118, 3926–3940. doi:10.1002/bit.27868

Maiti, N. C., Apetri, M. M., Zagorski, M. G., Carey, P. R., and Anderson, V. E. (2004). Raman Spectroscopic Characterization of Secondary Structure in Natively Unfolded Proteins: α-Synuclein. *Journal of the American Chemical Society* 126, 2399–2408. doi:10.1021/ja0356176

Rygula, A., Majzner, K., Marzec, K. M., Kaczor, A., Pilarczyk, M., and Baranska, M. (2013). Raman spectroscopy of proteins: A review. *Journal of Raman Spectroscopy* 44, 1061–1076. doi:10.1002/jrs.4335
